# Activation of human endogenous retroviruses by Sox proteins induces cell apoptosis via the caspase-3 pathway

**DOI:** 10.3389/fmicb.2025.1604022

**Published:** 2025-09-04

**Authors:** Md. Jakir Hossain, Nami Monde, Hiroyuki Sasaki, Perpetual Nyame, Wright Andrews Ofotsu Amesimeku, Hiromi Terasawa, Sojiro Matsumura, Takeshi Matsui, Hiroyasu Tsutsuki, Yosuke Maeda, Tomohiro Sawa, Kazuaki Monde

**Affiliations:** ^1^Department of Microbiology, Faculty of Life Sciences, Kumamoto University, Kumamoto, Japan; ^2^Department of Occupational Therapy, School of Rehabilitation, Tokyo Professional University of Health Sciences, Tokyo, Japan; ^3^Laboratory for Evolutionary Cell Biology of the Skin, School of Bioscience and Biotechnology, Tokyo University of Technology, Tokyo, Japan; ^4^Department of Nursing, Kibi International University, Okayama, Japan; ^5^Collaboration Unit for Infection, Joint Research Center for Human Retrovirus Infection, Kumamoto University, Kumamoto, Japan

**Keywords:** HERVs, Sox, retrotransposition, apoptosis, electron microscopy

## Abstract

Human endogenous retroviruses (HERVs) were domesticated millions of years ago as ancestral relics through germline infections and have become part of the human genome (8.3%). Over time, HERVs lost their innate ability to become virulent. We have previously reported that the transcription factor Sox2 is critical for human endogenous retrovirus-K (HERV-K) LTR5H activation and transposition in induced pluripotent stem cells. In the present study, we identified HERV-K LTR5H and LTR5B activation following Sox overexpression. In addition, we found that HERV-K Gag localized in the plasma membrane and that virus-like particles were released from Sox-expressing cells. Notably, a deformed nucleus was induced by cleaved caspase-3 in the HERV-K Gag-expressing cells. The caspase-3 inhibitors increased the number of HERV-K Gag-expressing cells by inhibiting the apoptotic pathway. Furthermore, retrotransposition of HERV-K was significantly enhanced in Sox2-expressing cells treated with caspase-3 inhibitors. Taken together, these results indicate that several Sox proteins increase HERV-K expression with cleaved caspase-3, suggesting that induction of the cell apoptotic pathway prevents genome impairment by HERV-K expression and retrotransposition.

## Introduction

Retroviruses represent all known viruses that exist endogenously or exogenously (Aiewsakun and Katzourakis, 2015; Horie et al., 2010; Johnson, 2015). When a retrovirus infects germline cells and is passed on to the offspring, it gradually becomes an endogenized remnant, such as human endogenous retroviruses (HERVs), which occupy 8.3% of the human genome (Duan et al., 2021). HERVs are epigenetically silenced via cellular mechanisms such as those that involve TRIM28 (Srinivasachar Badarinarayan and Sauter, 2022). However, HERV-transcribed genes are present in reproductive tissues and somatic tissues, such as the heart, lungs, intestine, brain, placenta, and embryonic tissues (Adamson and Freed, 2007; Grow et al., 2015; Mallet et al., 2004; Prudhomme et al., 2004). In addition, they are expressed in many cells, including aberrant cells, especially in cancer cells (Flockerzi et al., 2008; Frank et al., 2008), and in patients with rheumatoid arthritis (Freimanis et al., 2010; Reynier et al., 2009), schizophrenia (Dickerson et al., 2008; Frank et al., 2005), and amyotrophic lateral sclerosis (Douville et al., 2011; Guo et al., 2018). Activated ERVs and their gene products are potential drivers of abnormalities, such as neurological disorders, cellular senescence, oncogenesis, and autoimmune diseases (Antony et al., 2011; Balada et al., 2009; Groger et al., 2021; Liu S. et al., 2023).

The open reading frame (ORF) of retroviruses is usually transcribed from its 5' long terminal repeat (LTR) via transcription factor (TFs) binding (Garcia-Sanchez and Marques-Garcia, 2016; Liu and Eiden, 2011; Prudhomme et al., 2004; Ruda et al., 2004; Yu et al., 2013). Additionally, ERV can recombine their 5' and 3' LTRs to generate a solo LTR, which can modulate neighboring genes (Fuentes et al., 2018; Hossain et al., 2024; Hughes and Coffin, 2004). Various transcription factor-binding motifs are present in the HERV LTR to regulate their transcription (Bannert et al., 2018; Doucet-O'hare et al., 2021). We previously reported that Sox2 is a crucial transcription factor that initiates the transcription of HERV-K LTR5Hs and LTR5B (Monde et al., 2022). However, whether other Sox proteins bind to HERV LTRs remains unknown.

Sox proteins are classified into eight classes (A-H) based on their structure and gene expression profiles. SoxB is further classified into SoxB1 (Sox1, Sox2, and Sox3) and SoxB2 (Sox14 and Sox21). Sox4, Sox11, and Sox12 are SoxC. SoxD is further classified into Sox5, Sox6, and Sox13. SoxE can be classified into Sox8, Sox9, and Sox10. Sox7, Sox17, and Sox18 are classified as SoxF. SoxG and H are classified into Sox15 and Sox30, respectively (Schepers et al., 2002). Sox proteins are expressed in different healthy tissues: Sox1 in the eye (Venere et al., 2012); Sox2 in stem cells (Zhang and Cui, 2014); Sox3 in neural cells (Nitta et al., 2006); Sox4 in pancreatic cells (Xu et al., 2015); Sox9 in bone and cartilage (Bi et al., 1999); Sox10 in melanocytes of skin (Capparelli et al., 2022); and Sox17 in renal cells of the kidney and urinary tract (Gimelli et al., 2010). Conversely, Sox proteins are also expressed in malfunctioning tissues. Sox2, Sox3, Sox4, and Sox9 potentially contribute to tumor proliferation, invasion, and malignancy in lung cancer (Grimm et al., 2020). In breast cancer, Sox2, Sox4, Sox9, and Sox10 may be associated with cancer stem cell properties and resistance to therapy (Grimm et al., 2020). Sox4, Sox9, and Sox17 may play roles in cancer progression and metastasis in hepatocellular carcinoma. Sox2 may contribute to abnormal cell proliferation, dysregulated differentiation, or impaired tissue function in sarcoma, systemic lupus erythematosus, T-cell leukemia, and neurological disease, reflecting its role as a key transcription factor in maintaining stemness and regulating cell fate (Ahmed et al., 2022; Grimm et al., 2020; Liu X. et al., 2023; Madill et al., 2017; Mercurio et al., 2022). In ALS, the expression of Sox2 and Sox10 may be involved in neural cell stress responses or attempts at regenerative processes, highlighting a possible link between Sox factors and neurodegenerative disease mechanisms (Haag et al., 2008). Sox4 and Sox13 may affect pancreatic β-cell function and immune responses in type 1 diabetes (Kasimiotis et al., 2000; Wilson et al., 2005). Therefore, elucidating the mechanisms of transactivation may provide important insights into HERV-related diseases.

HERV proteins play several physiological roles in cells. For example, HERV-K Rec expression protects the host from infection by exogenous viruses by inducing IFITM expression (Grow et al., 2015). HERV-K Gag interferes with the particle formation of retroviruses, such as HIV-1, thereby reducing viral release and infectivity (Monde et al., 2012, 2017). In contrast, Rec accelerates cancer progression and induces cell death (Galli et al., 2005; Tavakolian et al., 2019). GagProPol also causes the retrotransposition of viral genes in the host genome (Monde et al., 2022).

In the present study, we found that Sox proteins activated HERV-K transcription, and then HERV-K expression induced cell death via the apoptotic pathway. Therefore, the newly integrated HERV-K into the genome via retrotransposition was eventually eliminated through cell apoptosis. Hence, our findings contribute to understanding the physiological roles of Sox-activated HERVs.

## Method

### Plasmids

Different HERV LTRs were amplified from the total DNA of NCCIT cells (Teratocarcinoma cells) using LTR-specific primers and templates. The primer sequences are listed in Supplementary Table 1. The isolated LTRs were inserted into the pHERV-K LTR Venus/luciferase vector. Additionally, LTR5Hs was inserted in the vector where Venus is in reverse orientation (3' to 5'). A FLAG peptide sequence was fused to the C-terminus of each Sox in the pMXs plasmid (Addgene, MA, USA), and primer information is provided in Supplementary Table 1. pRL-TK was obtained from Addgene. pmCherry-N1 was obtained from Takara clontech. Full-length HERV-K_CON_ and CHKCP were kindly provided by Paul Bieniasz (Lee and Bieniasz, 2007). pCMV-K/GagProPol/inNluc was derived from CHKCP as reported previously (Monde et al., 2022). pHERV-K/GagProPol/inNluc encoded the intact HERV-K_CON_ 5'LTR instead of CMV promoter.

### Cells

The teratocarcinoma cell line NCCIT was obtained from American Type Culture Collection (VA, USA). The cells were cultured in Roswell Park Memorial Institute medium (Thermo Fisher Scientific, Waltham, MA, USA) supplemented with 10% fetal bovine serum (FBS; Gibco, NY, USA), 1% GlutaMAX (Thermo Fisher Scientific), 1 mM sodium pyruvate (Thermo Fisher Scientific), and 1% penicillin/streptomycin (P/S). NCCIT/KOSOX2 cells were established as described previously (Monde et al., 2022). HeLa and 293T cells were cultured in Dulbecco's modified Eagle's medium (Sigma-Aldrich, St. Louis, MO, USA) supplemented with 5% FBS, 1% L-glutamine (Thermo Fisher Scientific), and 1% P/S (Thermo Fisher Scientific).

### Measurement of dual-luciferase luminescence

HeLa cells were seeded into 48 well plates. The HERVs-LTR firefly luciferase construct (0.15 μg) was cotransfected with Renilla luciferase plasmids (pRL-TK) (0.02 μg) and all Sox plasmids (0.05 μg) separately into HeLa cells. The cells were lysed with passive lysis buffer (Promega, WI, USA) 48 h post-transfection. Cell lysates were mixed with substrates using a dual-luciferase reporter assay system (Promega) according to the manufacturer's instructions. Firefly luciferase activity was measured using GloMax EXPLORER (Promega). Renilla luciferase activity was measured using the same luminometer after adding the Stop & Glo reagent. The activity of firefly luciferase activity was divided by that of the internal control Renilla luciferase.

### Flow cytometry analysis

HeLa cells were seeded into 12 well plates. The HERVs LTR Venus (0.6 μg) and mCherry (0.06 μg) plasmids were cotransfected with each Sox (0.05 μg) into HeLa cells. Two days post-transfection, the cells were fixed with 4% paraformaldehyde (PFA) (Wako Chemicals, Japan) and permeabilized using 0.1% Triton X-100 (Wako Chemicals) for 2 min. After washing with phosphate-buffered saline (PBS), the cells were blocked with 3% bovine serum albumin (BSA; Nacalai Tesque, Kyoto, Japan) in PBS. The cells were incubated for 1 h with anti-cleaved caspase-3 rabbit monoclonal antibody (1:100; Cell Signaling Technology, MA, USA), and after subsequent washing, anti-rabbit IgG Alexa Fluor 488 (1:100; Life Technologies) in 3% BSA/PBS was added for another 1 h. Anti-HERV-K capsid mouse monoclonal antibody (1:100; AUSTRAL Biologicals, CA, USA) in 3% BSA/PBS was added for 1 h. Subsequently, the cells were washed and incubated with anti-mouse IgG Alexa Fluor 647 (1:100; Life Technologies, CA, USA) in 3% BSA/PBS. Flow cytometry of all experimental samples was performed using BD FACSCalibur (BD, NJ, USA) to determine fluorescence-positive cells. Venus- and mCherry-positive cells were detected using the FL1 and FL3 detectors, respectively, whereas Alexa Fluor 488 and Alexa Fluor 647 were detected using FL1 and FL4, respectively.

NCCIT, HeLa, and 293T cells were treated with the antiapoptotic drugs Z-DEVD-FMK (10 μM; Selleck Chemicals, TX, USA) and Ac-DEVD-CHO (2 μM; Selleck) for 2 h, after which the medium was replaced with a fresh medium. Subsequently, pCMV-K/GagProPol, pCMV-K/GagProPolΔRec, and Sox2 were transfected. At 48 h post-transfection, 1 × 10^5^ cells were fixed with 4% PFA and permeabilized using 0.1% Triton X-100. Next, an anti-HERV-K capsid mouse monoclonal antibody was added, followed by anti-mouse IgG Alexa Fluor 647, as described above. After three times consecutive washes with 5% BSA/PBS, an anti-cleaved caspase-3 rabbit monoclonal antibody was added and incubated overnight. The following day, anti-rabbit IgG Alexa Flour 488 was added. Stained cells were analyzed via flow cytometry (BD FACSCalibur).

### Chromatin immunoprecipitation (ChIP) assay

Sox-FLAG overexpressing HeLa cells (4 × 10^7^ cells) were harvested and fixed in 1% formaldehyde. After fixation, the cells were lysed with 20% NP-40, 10 mM HEPES-KOH (pH 7.9), 0.5 mM dithiothreitol (DTT), 10 mM KCl, 1.5 mM MgCl_2_, and a protease inhibitor. Subsequently, the samples were digested with micrococcal nuclease (37 °C, 20 min). Samples were further treated with 50 mM Tris-HCl (pH 8.1), 1.5 mM ethylenediaminetetraacetic acid, 10% sodium dodecyl sulfate (SDS), and a cocktail reagent containing a protease inhibitor and subsequently fragmented via sonication (Level-4, 20s for 10 times repeatedly). Sox-FLAG protein-bound DNA complexes were precipitated after overnight incubation at 4 °C with an anti-FLAG antibody (1:100; BioLegend, CA, USA). Then, ChIP-grade protein-G magnetic beads (Cell Signaling Technology) were added to the samples and incubated for 2 h at 4 °C using a rotator. After consecutive washes with low- and high-salt buffers, the samples were eluted with elution buffer (1 M NaHCO_3_, 10% SDS, and 1 M DTT). The eluted samples were subjected to phenol/chloroform extraction, followed by ethanol precipitation. After drying, the samples were dissolved in water.

### Reverse transcription and quantitative-PCR analysis

For reverse transcription PCR (RT-PCR) and reverse transcription quantitative PCR (RT-qPCR), the total mRNA of HeLa and NCCIT cells was extracted using an RNeasy Mini Kit (Qiagen, Germany) and then reverse transcribed using random hexamer and murine leukemia virus reverse transcriptase in SuperScript™ IV (Thermo Fisher Scientific). HERV-K *gag* DNA was amplified using the primers listed in Supplementary Table 1. The harvested ChIP-DNA was analyzed via PCR (TaKaRa Bio Inc., Shiga, Japan) using HERV-K LTR primers (Supplementary Table 1). Quantitative PCR was performed on an using an ABI 7900HT Fast Real-Time PCR System (Applied Biosystems, MA, USA) using KAPA SYBR FAST (Roche, Basel, Switzerland) according to the manufacturer's instructions. The housekeeping gene glyceraldehyde-3-phosphate dehydrogenase (GAPDH) was amplified using the primers listed in Supplementary Table 1.

### Western blot analysis

Each Sox and Sox-FLAG plasmid was transfected into 293T and HeLa cells, respectively. After 48 h of transfection, the cells and their supernatants were harvested. The viruses/Sox proteins in the supernatant were pelleted via centrifugation at 13,200 × *g* for 1 h. The samples were lysed with 1% Triton X-100 lysis buffer [50 mM Tris-HCl (pH 7.5), 10 mM iodoacetamide, 300 mM NaCl, and a protease inhibitor cocktail (Roche)]. The samples were then treated with 2 × SDS sample buffer. The western blot analysis was performed using anti-HERV-K capsid protein (1:100; AUSTRAL Biologicals), anti-FLAG mouse monoclonal antibody (1:100; Wako Chemicals), and anti-GAPDH mouse monoclonal antibody (1:2,000; Sigma-Aldrich). Horseradish peroxidase (HRP)-conjugated anti-mouse IgG antibody (1:2,000; Jackson ImmunoResearch, PA, USA) was used as the secondary antibody. HRP-conjugated secondary antibodies were detected using Chemi-Lumi One L (Nacalai Tesque).

### Confocal microscopy

NCCIT and HeLa cells were seeded in eight-well chamber slides (WATSON Bio Lab, CA, USA) at a density of 5.0 × 10^4^ cells/well. The chamber slide wells were coated with collagen (KOKEN Co., Ltd., Tokyo, Japan) before cell seeding. HeLa cells were cotransfected with pMXs-Sox2, pCMV-K/GagProPolΔRec, and pCMV-K/GagProPol independently, as described above, and incubated for 48 h. The cells were fixed with 4% PFA (Wako Chemicals) for 30 min at 4 °C. The cells were then permeabilized with 0.1% Triton-X-100 for 2 min. After adding 0.1 M glycine for 10 min, the cells were blocked via treatment with 3% BSA solution for 30 min at room temperature. Subsequently, the cells were incubated with an anti-HERV-K capsid mouse monoclonal antibody (1:100; AUSTRAL Biologicals) for 1 h at 4 °C. After washing, the cells were then incubated with anti-mouse Alexa Flour 488 (1:100; Life Technologies) for 1 h at 4 °C. In the case of apoptotic cells staining, the anti-cleaved caspase-3 rabbit monoclonal antibody (1:100; Cell Signaling Technology, MA, USA), followed by anti-rabbit IgG Alexa Fluor 488 (1:100; Life Technologies) as the secondary antibody and anti-HERV-K capsid mouse monoclonal antibody (1:100; AUSTRAL Biologicals) followed by anti-mouse IgG Alexa Flour-546 (1:100; Life Technologies) was added. Antibodies were incubated for 1 h. Finally, the samples were stained with 4',6-diamidino-2-phenylindole (DAPI) and incubated for 5 min at room temperature. The samples were mounted using a fluorescence mounting medium (Dako, Denmark) and were imaged (15–20 fields) using an LSM 700 laser-scanning confocal microscope (Zeiss, Germany).

### HERV-K retrotransposition assay

The HERV-K retrotransposition assay was performed as described previously (Monde et al., 2022). Briefly, pCMV-K/GagProPol/inNluc and pHERV-K_CON_/GagProPol/inNluc were transfected into 293T cells. Nanoluciferase activity in the cells was measured using a Nano-Glo Luciferase Assay Reagent (Promega).

### Electron microscopy

For transmission electron microscopy, pelleted viruses were harvested from the cell supernatant of Sox2-transfected HeLa or NCCIT cells. Pelleted viruses were fixed with 2% glutaraldehyde (TAAB Laboratories Equipment, Aldermaston, England) and 1% osmium tetroxide (TAAB Laboratories Equipment) and dehydrated by a graded series of 50, 70, 80, 90, 95, and 99.5% ethanol, respectively. The samples were then embedded in Epon812 resin (TAAB Laboratories Equipment). Ultrathin sections on copper grids (Nisshin EM) were stained with Mayer's hematoxylin solution (Sigma-Aldrich) and lead citrate (Sigma-Aldrich) (Sasaki et al., 2022). The stained samples were observed and recorded using a Hitachi 7600 transmission electron microscope (Hitachi High-Technologies, Tokyo, Japan) at 80 kV.

For scanning electron microscopy, NCCIT cells, NCCIT/KOSOX2 cells, and 293T were seeded on top of the cover slides. 293T cells were transfected with pCMV-K/GagProPolΔRec/inNluc, pCMV-K/GagProPol/inNluc, and Sox2. 48 h post-transfection, the cells on the cover slides were doubly fixed with 2% glutaraldehyde (TAAB Laboratories Equipment) and 1% osmium tetroxide (TAAB Laboratories Equipment) and then dehydrated by a graded series of ethanol. Samples were immersed in hexamethyldisilazane (Sigma-Aldrich), air dried, and ion sputter coated with platinum. Images were taken with a JEOL JSM-7200F scanning electron microscope (JEOL, Tokyo, Japan) at 5 kV.

### Statistical analysis

Statistical analysis was performed to determine the significance of differences between the experimental and control groups. The results were analyzed using a two-tailed *t*-test (Excel Ver.16.0.18827.20102).

### Safety precautions

All experiments involving HERV-K were performed in accordance with BSL-3 safety guidelines, as the virus-like particles (VLPs) derived from HERV-K are considered non-infectious and non-pathogenic. All instruments and disposable materials exposed to VLPs were decontaminated using appropriate disinfectants or autoclaved to ensure biosafety.

## Results

### Sox proteins activate HERV-K LTR5H and LTR5B transcription

**Figure 1:**
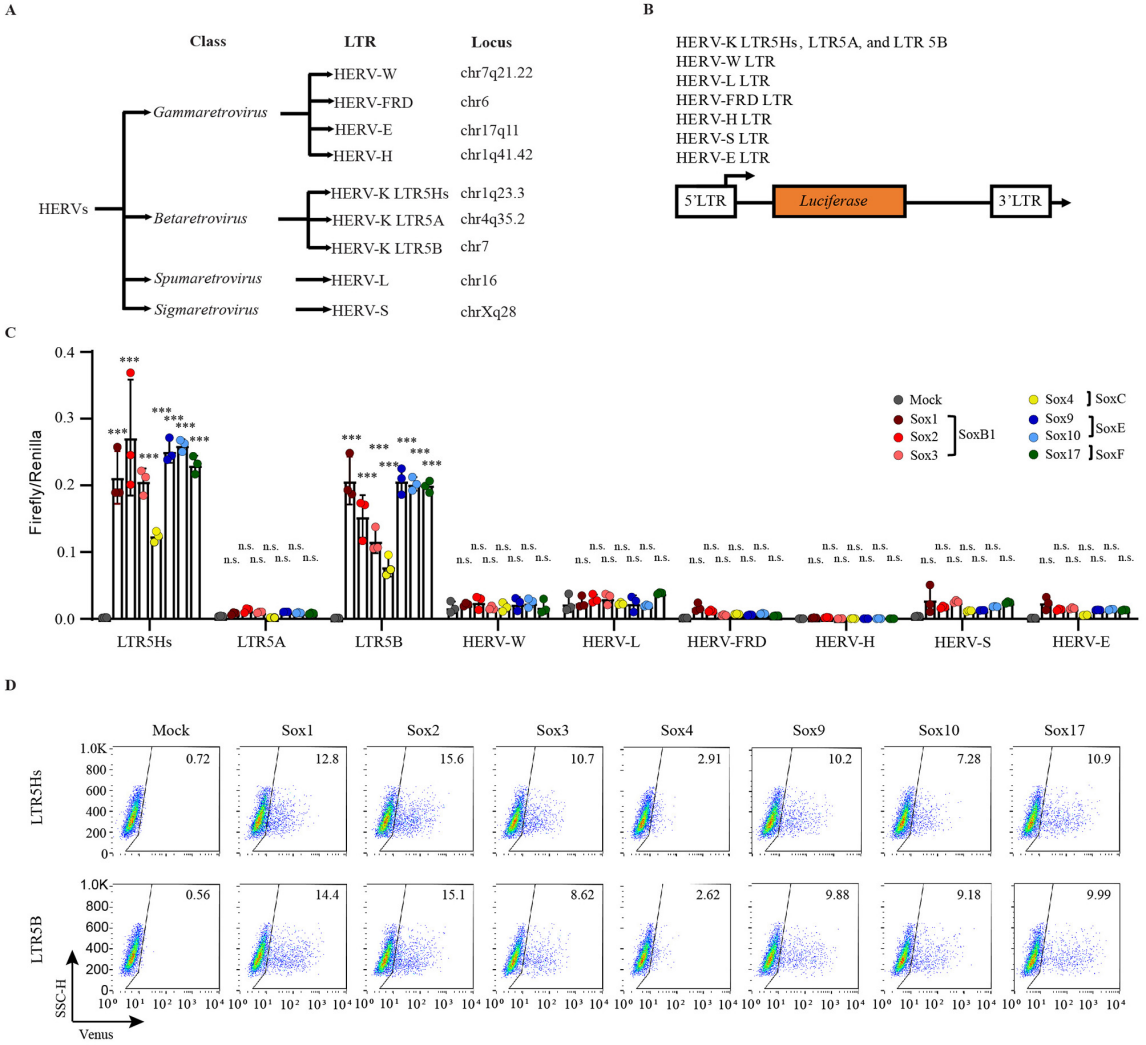
Sox proteins activated HERV-K LTR5Hs and LTR5B in HeLa cells. **(A)** HERVs LTR were isolated from NCCIT cells, and the locations were determined based on sequence alignment using BLAST search (Prudhomme et al., 2004; Shiroma et al., 2001; Subramanian et al., 2011; Vargas et al., 2009; Wentzensen et al., 2007). **(B)** pHERV-K LTR5Hs, LTR5A, and LTR5B, pHERV-W, L, FRD, H, S, and E LTR-driven firefly luciferase plasmids were constructed. **(C)** pMXs-Sox1-4, 9, 10, and 17, pRT-TK, and pHERVs LTR plasmids were cotransfected into HeLa cells. The firefly luciferase activity was divided by internal control renilla luciferase activity. For statistically significant analysis, the data from three independent experiments are shown as mean ± standard deviations. The P values were determined by the student's *t*-test. ***P < 0.0001; n.s., not significant. **(D)** The venus-positive cells were analyzed using flow cytometry.

Previously, we reported that HERV-K LTR5Hs were activated in Sox2-expressing teratocarcinoma cells (NCCIT cells) (Monde et al., 2022). Therefore, to investigate whether other HERV LTRs are activated by Sox2, we isolated them from the genome of NCCIT cells and identified their loci using the basic local alignment search tool (BLAST) search (Chen M. et al., 2024; Hurst and Magiorkinis, 2017; Schon et al., 2009; Figure 1A). The locations of the HERV loci were as follows: chr1q23.3 (HERV-K LTR5Hs), chr4q35.2 (LTR5A), chr7 (LTR5B), chr7q21.22 (HERV-W LTR), chr16 (HERV-L LTR), chr6 (HERV-FRD LTR), chr1q41.42 (HERV-H LTR), chrXq28 (HERV-S LTR), and chr17q11 (HERV-E LTR) (Figure 1A). Next, we designed constructs encoding *luciferase*/*venus* as reporter genes downstream of the isolated LTRs (Figure 1B). HeLa cells did not endogenously express Sox proteins (Chandimali et al., 2020) or Oct3/4 (Clemente-Perivan et al., 2020; Rijlaarsdam et al., 2011; Wang et al., 2013); thus, HERVs were not activated in HeLa cells (Figure 1C; gray circles). Upon Sox2 overexpression, HERV-K LTR5Hs and LTR5B became highly activated; however, no other HERV LTRs were observed (Figure 1C; red circles). Promo software (Farre et al., 2003; Messeguer et al., 2002) revealed several Sox2 binding motifs in each HERV LTR (Supplementary Figures 1A, B). Therefore, we hypothesized that HERV LTRs are activated by other Sox proteins because all Sox families specifically bind to CATTGT-like DNA sequence (Kraus et al., 2011; Remenyi et al., 2003). However, Sox proteins only activated HERV-K LTR5Hs and LTR5B, but not other HERV LTRs (Figure 1C). Interestingly, Sox4 activated HERV-K LTR5Hs and LTR5B to a lesser extent upon Sox4 overexpression in HeLa cells (Figures 1C, D, and Supplementary Figure 2). We confirmed that the FLAG-tag does not change the phenotype of Sox-driven HERV-K LTR5Hs activation (Supplementary Figure 3). The expression of FLAG-tagged Sox proteins was detected in HeLa cells (Supplementary Figure 4). On the other hand, all Sox proteins, including Sox4, showed similar HERV-K LTR5Hs activation from the antisense strand (Supplementary Figures 5A, B). These results suggest that, unlike other Sox proteins, Sox4 initiates transcription from LTRs predominantly in a single direction. In Sox2-knockout NCCIT cells, several Sox proteins activated only HERV-K LTR5Hs and LTR5B (Supplementary Figures 6A, B). Notably, HERV-L LTR was activated in the absence of Sox2 in NCCIT/KOSOX2 cells (Supplementary Figure 6B). Altogether, the lack of HERV LTR activation despite the presence of Sox-binding motifs suggests that other transcription factors are required for activation.

### Sox proteins bind to multiple sites on HERV-K LTR5H

**Figure 2:**
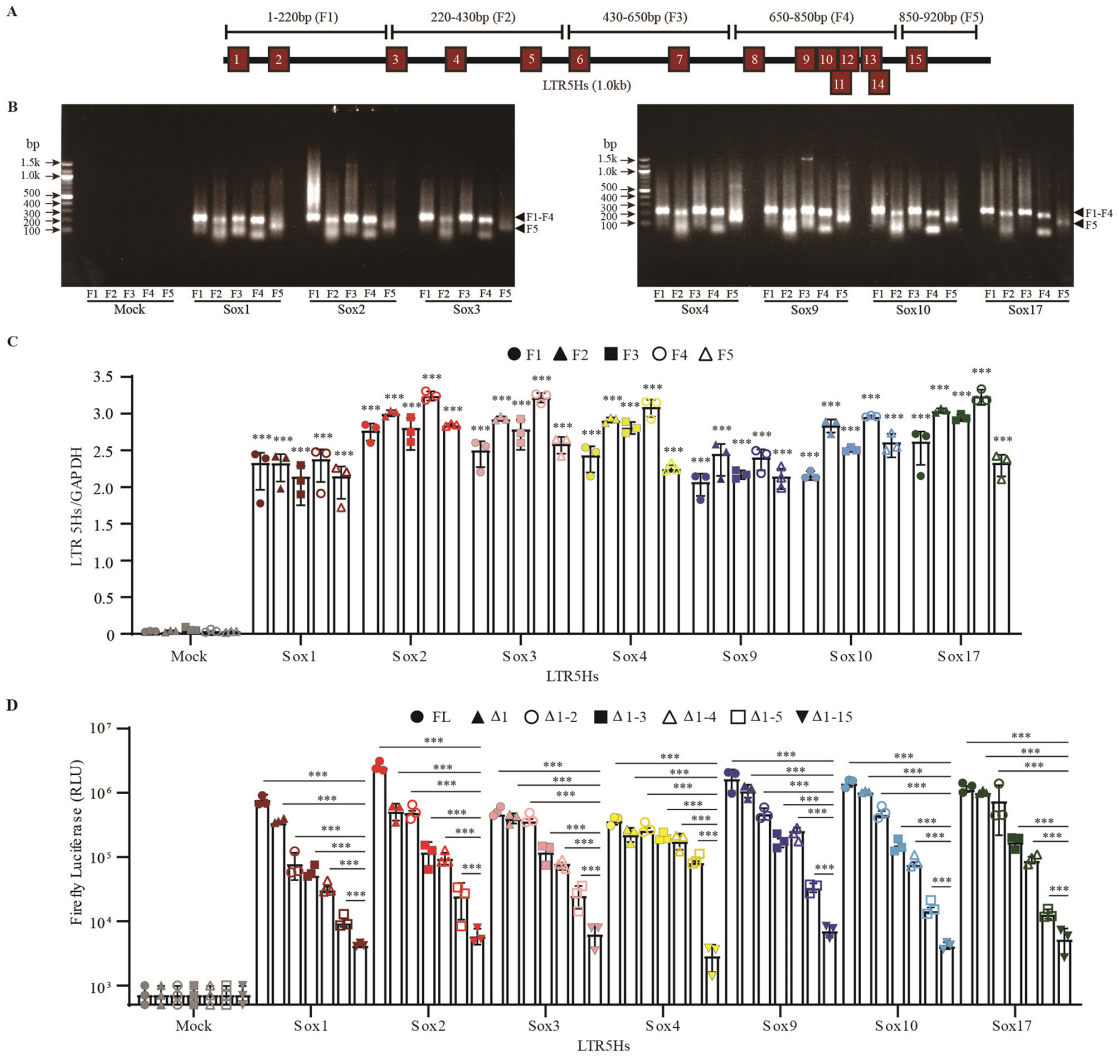
Sox proteins bound to multiple sites on HERV-K LTR. **(A)** Fifteen binding motifs were presented onto the HERV-K LTR5Hs based on Promo software and previous report (Monde et al., 2022). Five primers were designed, ranging in length from 220, 210, 220, 200, and 70 nucleotides, and designated as F1 to F5, respectively, to identify the desired amplicons. **(B)** Sox proteins-bound DNA was harvested by chromatin immunoprecipitation (ChIP) using an anti-FLAG antibody. F1 to F5 fragments were amplified using the precipitated DNA. **(C)** The amounts of F1 to F5 fragments were quantified using quantitative-PCR (qPCR). The copy numbers of F1 to F5 fragments were divided by GAPDH copy numbers. **(D)** Sox proteins binding motifs deletions (HERV-K LTR5Hs LTRΔ1, Δ1-2, Δ1-3, Δ1-4, Δ1-5, and Δ1-15) were cotransfected with each Sox proteins. The firefly luciferase activity was divided by internal control renilla luciferase activity. **(C, D)** For statistically significant analysis, the data from three independent experiments are shown as mean ± standard deviations. The P values of the results were determined by the student's *t*-test. ***P < 0.0001; n.s., not significant.

Based on the ChIP database and PROMO software, 15 different Sox binding motifs were present in the HERV-K LTR5Hs (Figure 2A). According to previous reports, all members of the Sox family have a similar binding motif that is located at 15 different positions of HERV-K LTR (Ghafoori et al., 2024; Hamilton et al., 2022; Harley et al., 1994). In this study, we sought to identify the predominant binding sites for each Sox. HeLa cells were transfected with Sox-FLAG, and Sox-bound DNA was harvested via ChIP using an anti-FLAG antibody. Five primer sets were designed to amplify the F1-F5 fragments (Figure 2A). Fragment F4, which covers nucleotide numbers 650 to 850, showed the highest LTR copy number, while fragments F1 and F5 showed the lowest LTR copy number in the case of each Sox (Figures 2B, C). Previously, we reported that a single deletion of Sox2 binding sites is not sufficient for the loss of HERV-K LTR5Hs activation (Monde et al., 2022). Consistent with a previous report, multiple deletions of the Sox-binding motifs reduced HERV-K LTR5H activation (Figure 2D). These results suggest that specific DNA elements—putative Sox-Binding sites (T/A T/AGTTT/A and A/T A/TCAAA/T)—are required for full activation by Sox proteins.

### Sox proteins induce HERV-K Gag expression and virus-like particle release

**Figure 3:**
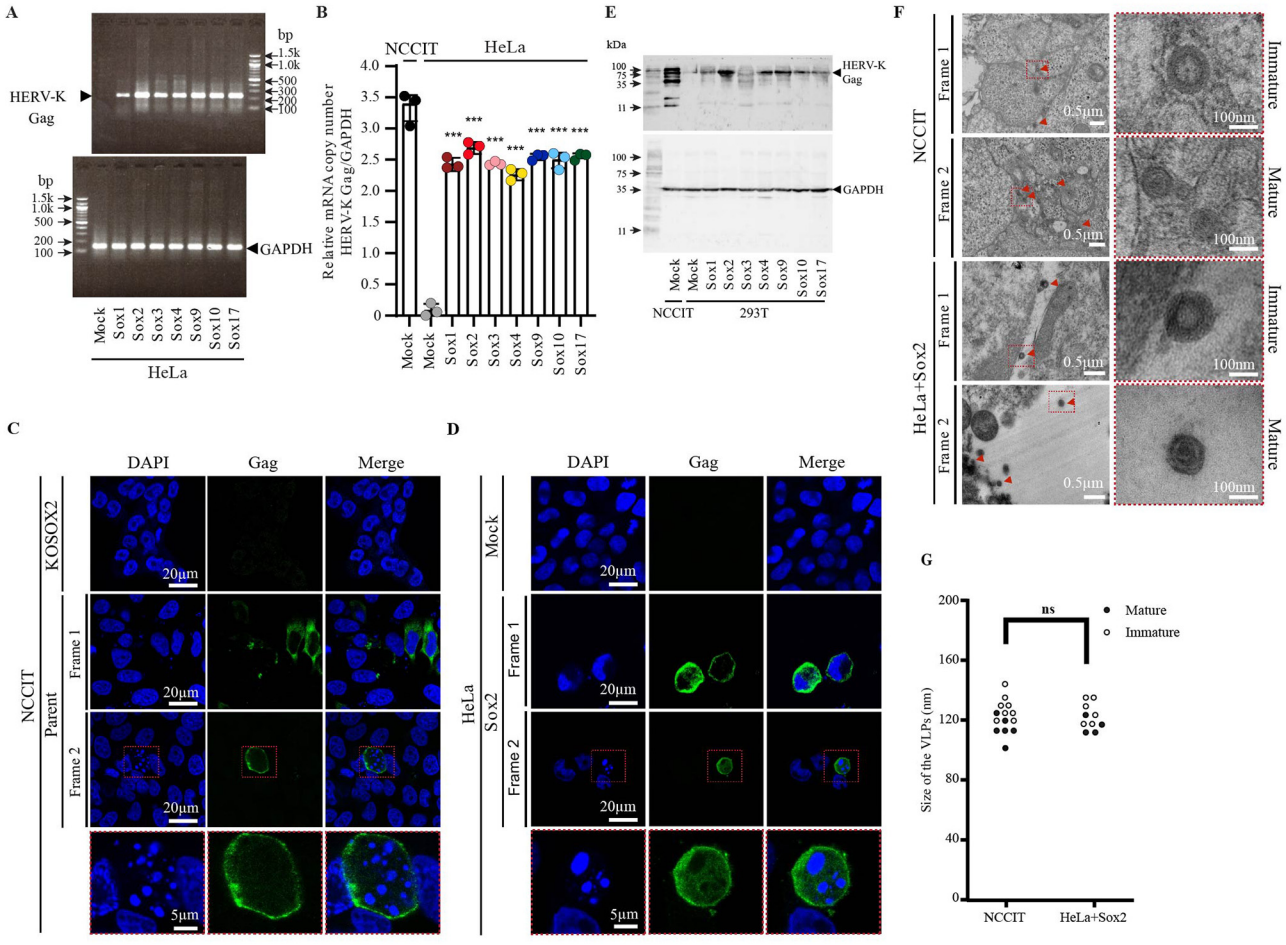
Sox proteins increased the HERV-K Gag expression and VLPs release. **(A)** Total mRNA was harvested from Sox overexpressing HeLa cells supernatant. HERV-K Gag and GAPDH mRNA were amplified by the reverse transcription PCR (RT-PCR). **(B)** The copy number of HERV-K Gag mRNA and GAPDH mRNA were quantified by RT-qPCR. The relative copy number of HERV-K Gag mRNA was calculated by dividing GAPDH mRNA. For statistically significant analysis, the data from three independent experiments are shown as mean ± standard deviations. According to the student's *t*-test, the *P* values were determined where ***P < 0.0001; n.s., not significant. **(C, D)** HERV-K Gag localization was observed by confocal microscopy. The green images indicated the HERV-K Gag localization and the blue images indicated the nucleus. The scale bar showed the size of the images (20 μm). **(E)** The viral proteins were harvested from the cell supernatants. Samples were subjected to western blot analysis by staining with anti-HERV-K capsid mouse monoclonal antibody. The housekeeping GAPDH in the cells was detected as an internal control. **(F)** Released mature and immature HERV-K VLPs from NCCIT and Sox2 overexpressing HeLa cells were observed by Electron microscope. The red arrowheads indicate the VLPs. The scale bar showed the size of the images. **(G)** The number and size of VLPs in each image were measured. White circles indicate immature VLPs, and black circles indicate mature VLPs.

To investigate whether Sox activated the transcription of endogenous HERV-K into the host genome, we measured the mRNA expression of HERV-K Gag upon Sox overexpression (Figures 3A, B). In NCCIT cells, which are Sox2-expressing teratocarcinoma cells, HERV-K Gag mRNA was constitutively expressed (Figure 3B). Similarly, in HeLa cells, all Sox proteins markedly increased HERV-K *gag* mRNA expression (Figures 3A, B). The majority of HERV-K *gag* in the human genome has multiple mutations, insertions, and deletions. However, as previously reported, subtypes HERV-K102, K103, K108b, K113, K115, and K119 possess an intact *gag* ORF in the human genome (Contreras-Galindo et al., 2017; Laderoute et al., 2015; Shin et al., 2013), and therefore have the potential to be functionally active in NCCIT and Sox2-expressing HeLa cells. We investigated whether the functional HERV-K Gag protein was expressed and formed virus-like particles (VLPs) in Sox-expressing cells. In NCCIT cells, HERV-K Gag was localized in the cytoplasm and plasma membrane (Figure 3C). In HeLa cells, HERV-K Gag was upregulated by the overexpression of Sox2 (Figure 3D). Notably, characteristic cellular morphological changes such as apoptosis and metaphase were observed in the HERV-K Gag-expressing cells (Figures 3C, D, Supplementary Figure 7). As previously reported, HERV-K Gag assembles and forms VLPs at the plasma membrane (Kraus et al., 2011) and is then released into cell supernatants. Both HERV-K mature (37 kDa) and immature (73 kDa) Gag were detected in the supernatant from NCCIT cells (Figure 3E). Unexpectedly, HERV-K Gag was undetectable or only faintly expressed in the supernatant of Soxs expressing HeLa cells (data not shown). Only HERV-K immature Gag was detected in the supernatants of Sox-expressing 293 T cells (Figure 3E). Additionally, both mature and immature forms of viruses were detected in Sox2-expressing HeLa cells via transmission electron microscopy. Consistent with previous reports (Krebs et al., 2023), the size of the HERV-K-like particles was approximately 120-140 nm (Figures 3F, G). Using scanning electron microscopy, many VLPs appeared on the NCCIT cells compared to NCCIT/KOSOX2 (Supplementary Figures 8A, C and Supplementary Table 2). A relatively large number of VLPs were observed on the pCMV-K/GagProPol-inNluc-transfected HeLa and Sox2-expressing HeLa cells compared to pCMV-K/GagProPolΔRec-inNluc-transfected HeLa (Supplementary Figures 8B, C and Supplementary Table 2). These results indicate that Sox2 overexpression induce HERV-K Gag expression and viral release from cells.

### HERV-K expression induces cell apoptosis

**Figure 4:**
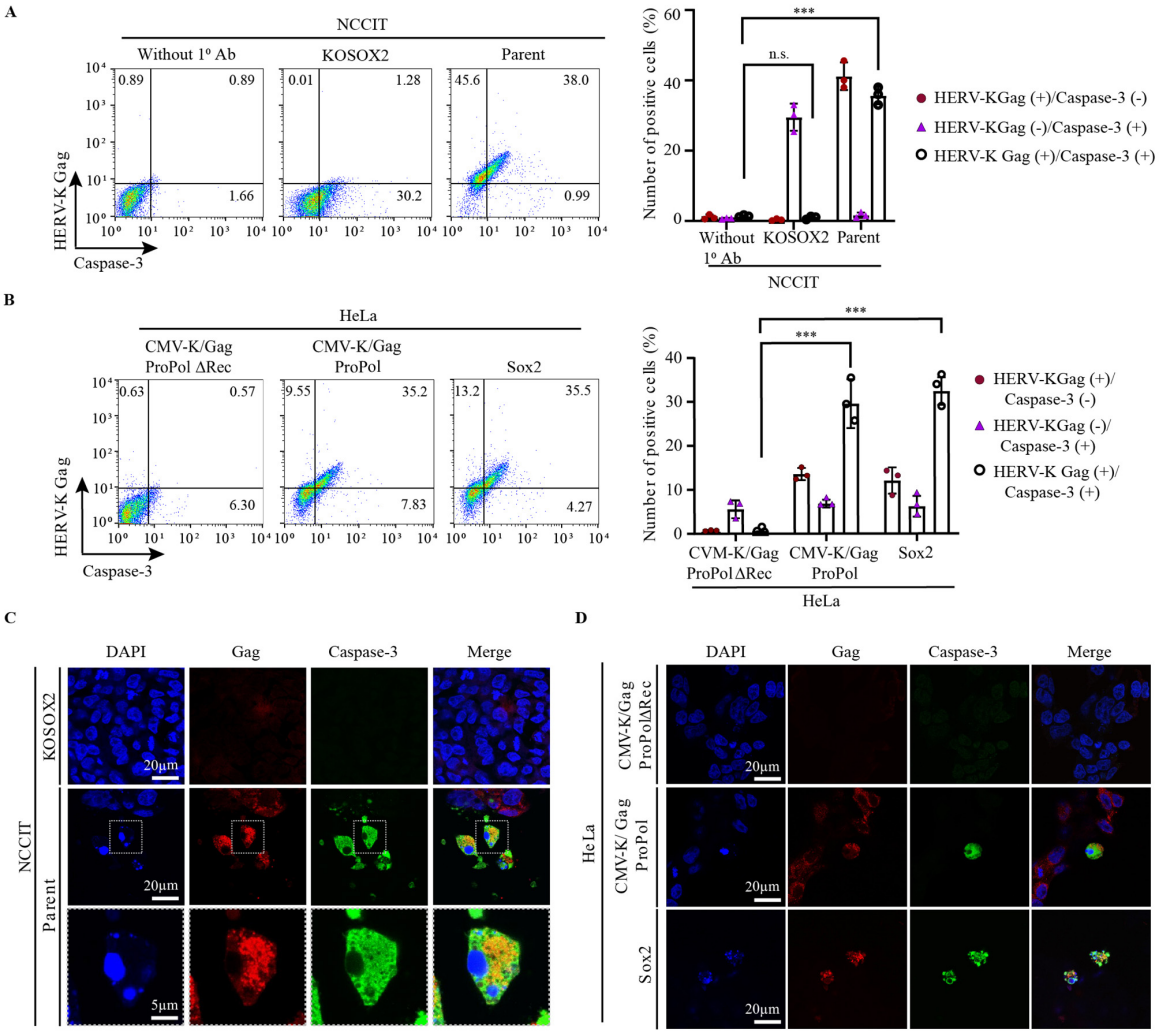
The HERV-K Gag co-localized with cleaved caspase-3 positive cells. **(A)** The parent NCCIT and NCCIT/KOSOX2 cells, and **(B)** CMV-K/GagProPolΔRec, CMV-K/GagProPol, and Sox2 overexpressing HeLa cells were stained with anti-HERV-K capsid antibody and anti-cleaved caspase-3 antibody. The NCCIT/KOSOX2 cells were stained with only a secondary antibody and designated as mock. For statistically significant analysis, the data from three independent experiments are shown as mean ± standard deviations. The P values were determined based on the student's *t*-test. ***P < 0.0001; n.s., not significant. **(C)** The parent NCCIT, NCCIT/KOSOX2, and **(D)** GagProPolΔRec, GagProPol, and Sox2 overexpressing HeLa cells were stained with antibodies and observed by confocal microscopy where red color representing HERV-K Gag and green for cleaved caspase-3 positive cells. The scale bar shows the size of images (20 μm).

The nuclei of HERV-K Gag-expressing cells were deformed (Figures 3C, D), and we hypothesized that HERV-K expression induces cell apoptosis. HERV-K Rec and Np9 are the key players in viral RNA transport and cell death (Chan S. M. et al., 2019; Chen J. et al., 2024; Hanke et al., 2013). We determined whether HERV-K expression induces cell death in Sox2-expressing cells by measuring the signal of cleaved caspase-3, an active enzyme in apoptotic cells, in HERV-K Gag-expressing cells (Brentnall et al., 2013). In NCCIT cells, a relatively high proportion (38%) of HERV-K Gag-expressing cells were positive for cleaved caspase-3 (Figure 4A). The size and morphology of the cells changed in HERV-K- and cleaved caspase-3-positive populations (Figures 4C, D and Supplementary Figure 9). Notably, NCCIT/KOSOX2 cells exhibited slow growth during passage (data not shown), and cleaved caspase-3 expression was elevated (Figure 4A and Supplementary Figure 9), possibly reflecting cellular stress or apoptosis associated with the loss of Sox2. Consistently, Sox2 induced the expression of both HERV-K Gag and cleaved caspase-3 in HeLa cells (Figure 4B). In addition, the number of cleaved caspase-3-positive cells were 6.3-fold increased by the overexpression of CMV-K/GagProPol in HeLa cells (Figure 4B). Furthermore, the nuclei were abnormal in cleaved caspase-3-expressing NCCIT and HeLa cells (Figures 4C, D). In Sox2-expressing HeLa cells, apoptotic cells appeared with many VLPs on the cell surface (Supplementary Figure 8B). These results indicate that HERV-K expression induces cleaved caspase-3 expression and cell apoptosis.

### Apoptosis inhibitors increase HERV-K expression and HERV-K transposition

**Figure 5:**
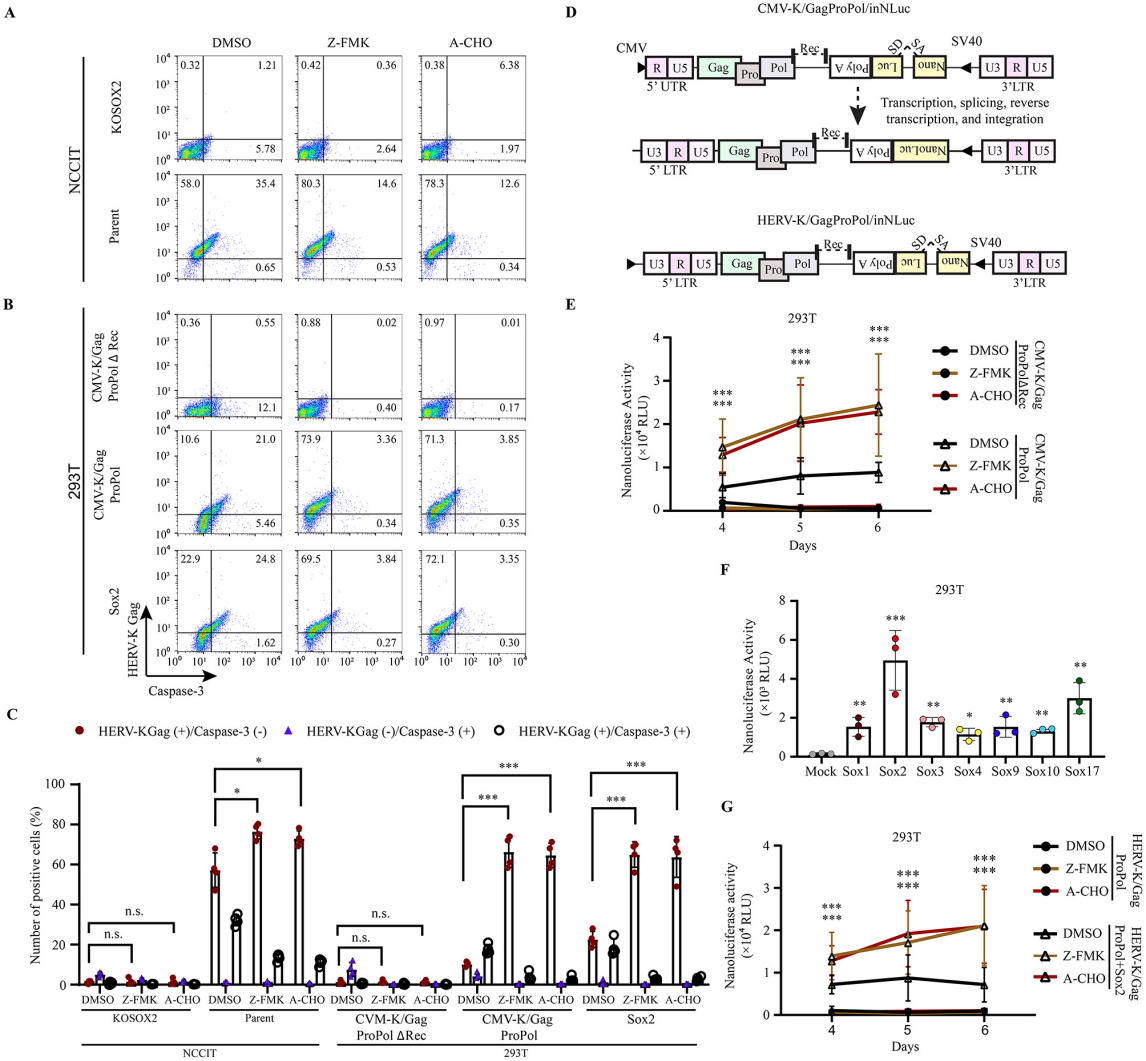
Apoptosis inhibition increased the HERV-K Gag expression, thereby transposition activity. **(A, B)** NCCIT cells and 293T cells were given Z-DEVD-FMK (Z-FMK) and Ac-DEVD-CHO (A-CHO) drugs, and 293T cells were transfected with pCMV-K/GagProPolΔRec, pCMV-K/GagProPol, and Sox2. The expression of HERV-K Gag with caspase-3 in the presence of drugs was compared with DMSO-treated cells. **(C)** Graphs represent the summarized flow cytometry results based on three independent day experiments. **(D)** A schematic diagram shows the order of the genetic information carrying the *nanoluciferase* gene with splicing donor and acceptor (SA/SD) to measure the HERV-K retrotransposition. **(E)** The nanoluciferase activity was quantitated from day 4 to day 6 in the presence of caspase-3 inhibitor drugs (Z-DEVD-FMK and Ac-DEVD-CHO). **(F)** LTR-driven HERV-K GagProPol was cotransfected with each of Sox in 293T cells. The nanoluciferase activity was measured on day 4. **(G)** The nanoluciferase activity was monitored from day 4 to day 6 in the Sox2-expressing 293T cells in the presence of caspase-3 inhibitor drugs (Z-DEVD-FMK and Ac-DEVD-CHO). **(C, E-G)** For statistically significant analysis, the data from three independent experiments are shown as mean ± standard deviations. The P values were determined based on the student's *t*-test. Where, *P < 0.01; **P < 0.001; ***P < 0.0001; n.s., not significant.

Previously, we reported that HERV-K in the host genome possesses retrotransposon activity. However, retrotransposition is rare, and the cells disappear after long-term culturing in iPSCs (Monde et al., 2022). It has been reported that HIV-1 infection induces caspase-3 activation, leading to apoptosis in infected cells (Cicala et al., 2000). Therefore, we hypothesized that apoptosis inhibitors would continuously increase HERV-K retrotransposition. First, we examined the expression of HERV-K Gag and cleaved caspase-3 in the presence of caspase-3 inhibitors (Figures 5A–C). These inhibitors (Z-DVED-FMK and Ac-DVED-CHO) broadly bind to caspases. They have no toxicity at 10 μM and 2 μM, respectively (Supplementary Figure 10). The percentage of HERV-K Gag- and Sox2-expressing 293T cells positive for cleaved caspase-3 was 24.8%. However, treatment with caspase-3 inhibitors Z-FMK and A-CHO reduced the number of these cells by 6.5-fold and 7.4-fold, respectively (Figure 5C). In addition, the number of HERV-K Gag-positive cells was significantly increased by treatment with caspase-3 inhibitors (Z-FMK: 1.5-fold, A-CHO: 1.6-fold; Figure 5C and Supplementary Figure 11). These results suggest that the number of HERV-K Gag-expressing cells decreases as a result of apoptosis.

To investigate the retrotransposition of HERV-K in the presence of caspase-3 inhibitors, we used a monitoring construct for the HERV-K retrotransposon (Figure 5D). Retrotransposition of HERV-K GagProPol, which encodes the CMV promoter at the 5' end, was significantly increased by caspase-3 inhibitors (Figure 5E). Using the CMV-K/GagProPol, retrotransposition was significantly increased in all Sox-expressing cells (Figure 5F). The retrotransposition of LTR5H GagProPol with Sox2 was significantly increased by caspase-3 inhibitors (Figure 5G). These results suggest that genome-impaired cells caused by HERV-K retrotransposition are selectively eliminated through the cell apoptotic pathway.

## Discussion

This study demonstrated that the Sox family could initiate the transcription of HERV-K LTR5Hs and LTR5B but not of other HERV LTRs, despite the presence of Sox-binding sites. The reason for the Sox-dependent activation of the relatively new HERV-K is unknown, but it may play a crucial role in early embryonic cells and other cells that express Sox. HERV-K Gag assembles in the plasma membrane of Sox-expressing cells to form VLPs. However, we could not clarify the physiological roles of the released VLP. Nonetheless, HERV-K retrotransposes and increases the genome size in Sox-expressing cells. Notably, the number of HERV-K-expressing cells markedly decreased via the apoptotic pathway. This study supports our previous finding that cells with newly integrated HERV-K grow slowly during long-term culture.

Sox proteins did not activate HERV-W, -L, -FRD, -H, or -E despite multiple Sox-binding motifs in LTRs (Figures 1C, D and Supplementary Figure 1). Induced pluripotent stem cells (iPSCs) express Sox2, Oct3/4, and Klf4. However, HERV-H is upregulated by Klf4, Oct3/4, and Nanog in iPSCs (Ohnuki et al., 2014). HERV-W is upregulated by c-Myb/HOXA5, Oct-1, GCM-a, Sp1, and Sp3 (Durnaoglu et al., 2021b; Lee et al., 2003; Yu et al., 2014). HERV-L is activated by HNF-1 (Durnaoglu et al., 2021a). HERV-E is activated by HIFs and NAFT1 (Wang et al., 2019). Therefore, these transcription factors might cooperate with Sox proteins for the HERV transcription. Of note, the expression of HERVs is precisely controlled by various transcription factors, suggesting that it is not a fossil remnant. They likely play essential roles in the long-term process of human adaptation via temporal and tissue-specific expression.

Sox proteins bind to the CATTGT-like DNA sequence that is located in HERV-K LTR5Hs and LTR5B. Sox4 initiated the transcription of the antisense strand from 3'LTR5Hs (Supplementary Figure 5). However, Sox4 poorly activated the transcription of sense strands from HERV-K 5'LTR5Hs and 5'LTR5B (Figures 1C, D). Although we cannot exclude the possibility that variation in Sox4 expression levels or transcript stability may contribute to the differential effects (Supplementary Figure 4; Beekman et al., 2012), the observed differences in HERV-K activation among Sox factors may also result from variations in the transcriptional cofactors that bind to Sox proteins. For example, Sox4 interacts with p53, β-catenin, plakoglobin, TCF4, KLF5, SMAD3, ERG, EV11, and NSD3, and forms complex for transcription (Boyd et al., 2006; David et al., 2016; Lai et al., 2011; Li et al., 2017; Pan et al., 2009; Sinner et al., 2007; Vervoort et al., 2018; Wang et al., 2014). These interacting cofactors with Sox4 might determine the direction of the transcription. Sox4 is expressed in pancreatic cells, lung cancer, breast cancer, and hepatocellular carcinoma (Grimm et al., 2020; Xu et al., 2015). In the future, it will be necessary to comprehensively analyze the cofactor expression and the expression pattern of genes near LTR5Hs and LTR5B in these cells.

Sox proteins are expressed in several healthy tissues and several cancer cells. HERV-K expression has been identified in germ cells and iPSCs, which express Sox2 (Grow et al., 2015; Monde et al., 2022). HERV-K expression has also been identified in several tumors, such as esophageal squamous cell carcinoma, melanoma, ovarian, prostate, glioblastoma, and lymph node tumor (Gonzalez-Cao et al., 2016; Nakamura et al., 2003; Zhao et al., 2011). In these cancer cells, some Sox proteins are upregulated, whereas others are downregulated (Grimm et al., 2020). However, the causal relationship between Sox and HERV activation in these cells remains unclear. Negative results other than LTR5Hs and LTR5B have been found regarding the causal relationship between the expression of ERVs and each other, leading to inconclusive findings (Sakashita et al., 2023; Sexton et al., 2022; Zhang et al., 2024). Regardless, in the field of cancer therapy, the anti-HERV-K Env antibody expressed in patients with cancer is helpful for the therapeutic efficacy of anti-PD-L1 (Ng et al., 2023). Although we have previously shown that HERV-K LTR5H and LTR5B are activated by Sox proteins (Monde et al., 2022), understanding the mechanisms by which the expression of HERVs is regulated is important to elucidate their physiological role and consider therapeutic approaches.

HERV-K expression induced cell apoptosis in NCCIT, HeLa, and 293T cells (Figure 4). HERV-K accessory protein Rec overexpression increases the levels of γH2AX, further damaging the host DNA, which leads to cell death (Chan C. M. et al., 2019; Chen J. et al., 2024). Replication stress due to the presence of a retrovirus causes cell death through ataxia telangiectasia and Rad3-related signal (Bester et al., 2013). In mouse models, an increase in ERV is a key factor in the development of replication stress in the hair follicular stem cells. Eventually, the cells die via apoptosis, leading to hair loss (Lyu et al., 2024). However, the mechanism of the cell apoptotic pathway by HERV expression has not yet been elucidated. In particular, it needs to be investigated in the future whether HERV-K Rec induces apoptosis or whether Gag-Pro-Pol expression and VLP production promote apoptosis. In addition, we have proven that HERV-K transposition is driven by Sox expression; however, the levels of newly integrated HERV-K gradually decrease during long-term culture (Monde et al., 2022). According to this finding, retrotransposition of HERV-K may be rare because caspase-3-mediated apoptosis induces cell death in HERV-K-expressing cells during long-term culture (Figures 5E, G). Thus, no genomic impairment due to HERV expression might be observed in cancer cells (Lopez-Moyado et al., 2019). Our findings strongly suggest that the decrease in the number of cells with newly integrated HERV was due to their elimination through apoptosis.

As previously reported, HERV-K expression is observed in two cells of the morula during early development (Grow et al., 2015). Subsequently, HERV-K must be silenced before implantation during embryonic development. TRIM28 and SETDB1, which are repressors, suppress ERV activation, allowing cell differentiation to proceed (Klimczak et al., 2017; Lyu et al., 2024; Rowe et al., 2013). In brief, HERV-K expression does not always induce cell death via the apoptotic pathway. However, some studies have shown that cells that are not needed die during embryonic development (Haanen and Vermes, 1996; Milligan and Schwartz, 1997), and our study may provide important insights into the regulation of cell death. Further investigation of cell apoptosis in iPSCs and germ cells is necessary to understand the relationship between HERV-K expression and cell death during early developmental stages.

We found the released VLP from Sox2-expressing cells (Figures 3E, F). In a previous study, a donor encoding a potentially infectious HERV-K was found in Africa (Wildschutte et al., 2016). However, the actual isolation of infectious HERV-K has not yet been reported because of the accumulation of several mutations (Dube et al., 2014; Muster et al., 2003; Srinivasachar Badarinarayan and Sauter, 2022). Notably, HERV-K type1 has a 292 bp deletion in the env gene. However, the Arc protein, which consists of ERV Gag, forms a VLP and is transmitted between synapses via the paracrine pathway (Fila et al., 2021; Sibarov et al., 2023). Thus, VLPs may propagate between specific and limited cells. However, the release of infectious HERV-K from HeLa, 293T, or teratocarcinoma cells has not yet been reported, and we found no data supporting viral propagation in our study (data not shown). The physiological role of released replication-incompetent viruses needs to be investigated in the future.

HERV is a retrovirus encoded in the genomes of all living organisms that benefit the host. However, the possibility that uncontrollable HERVs can harm the host cannot be ignored. There have been many potential studies on ERV-associated therapies. For example, in cancer therapy, the induction of anti-HERV-K Env antibodies with PD-L1 may be a topic of interest in the future, as described above (Ng et al., 2023). Anti-HERV compounds can be developed for senescence and hair loss therapy (Liu X. et al., 2023; Lyu et al., 2024). Therefore, clarifying the mechanism of HERV-K expression and the targeting of HERV-K-expressing cells may significantly influence the course of treatment.

## Data Availability

The original contributions presented in the study are included in the article/Supplementary material, further inquiries can be directed to the corresponding author.

## Supplementary material

Supplementary Figure 1 Sox binding motifs in LTRs. Except for HERV-S LTR, multiple Sox2 binding motifs were presented in HERV-K LTR5Hs, LTR5A, and LTR5B, HERV-W, L, FRD, H, and E LTR. Red lines indicate the Sox2-binding motifs.

Supplementary Figure 2 Sox proteins activated the HERV-K LTR5Hs and LTR5B in HeLa cells. Several pHERV LTR-Venus were cotransfected with the pMXs-Sox into HeLa cells. At 48 h post-transfection, HERV-K LTR5Hs, LTR5A, and LTR5B, HERV-W, L, FRD, H, S, and E LTR-driven venus positive signal was determined by flow cytometry.

Supplementary Figure 3 The Sox-FLAG proteins activated the HERV-K LTR5Hs activation. Sox-FLAG plasmids were cotransfected with HERV-K LTR5Hs-Venus plasmid into HeLa cells. At 48 h post-transfection, Venus-positive cells were detected by flow cytometry. For statistically significant analysis, the data from three independent experiments are shown as mean ± standard deviations. The *P* values were determined based on the student's *t*-test. Where, ^**^*P* < 0.001; ^***^*P* < 0.0001.

Supplementary Figure 4 The expression of Sox proteins in Sox-FLAG overexpressing HeLa cells. Sox-FLAG plasmids were transfected into HeLa cells. At 48 h post-transfection, the cell supernatants were collected. The molecular weight of each Sox was determined by staining with anti-FLAG antibody. Each molecular weight is shown in the right dot line box (Matsuba et al., 2002). The results are confirmed based on three independent experiments.

Supplementary Figure 5 Sox proteins activated HERV-K LTR5Hs from the antisense strand. **(A)** pHERV-K LTR5Hs 3'LTR-driven Venus plasmids were constructed. **(B)** pHERV-K LTR5Hs-Venus reverse orientation plasmid was cotransfected with pMXs-Sox into HeLa cells. At 48 h post-transfection, Venus-positive cells were detected by flow cytometry. For statistically significant analysis, the data from three independent experiments are shown as mean ± standard deviations. The *P* values were determined based on the student's *t*-test. Where, ^**^*P* < 0.001; ^***^*P* < 0.0001.

Supplementary Figure 6 HERV-K LTR5Hs and LTR5B were activated by Sox proteins in NCCIT/KOSOX2 cells. **(A)** Several pHERV LTR-Venus were cotransfected with the pMXs-Sox and pmCherry-N1 into NCCIT/KOSOX2. As internal control to normalize the transfection efficiency, the mCharry-expressing vector (pmCherry-N1) was cotransfected. The Venus and mCherry positive signals were determined by flow cytometry. **(B)** The Venus positive cell number was divided by the mCherry positive cell number. For statistically significant analysis, the data from three independent experiments are shown as mean ± standard deviations. According to the student's *t*-test, the *P* values were determined where ^***^*P* < 0.0001; n.s., not significant.

Supplementary Figure 7 The HERV-K Gag was highly expressed in NCCIT cells during cell division, as reported previously (Yamashita et al., 2020). The nucleus was stained with DAPI. The samples were subjected to stain with anti-HERV-K Gag antibody followed by Alexa Flour 488 and observed by confocal microscopy. The arrowhead indicates the expression of HERV-K Gag in dividing NCCIT cells (Frame 1, 2, and 3). The scale bar showed the size of the images.

Supplementary Figure 8 The number of VLPs were increased by Sox2 expression. **(A)** The VLP on the cell surface was observed by scanning electron microscopy in NCCIT/KOSOX2 and NCCIT cells. **(B)** The VLP on the cell surface was observed by scanning electron microscopy in pCMV-K/GagProPol?Rec, pCMV-K/GagProPolΔRec, and pMXs-Sox2-transfected 293T cells. The expanded images gated by yellow boxes show the right side. The scale bar showed the size of the images. The yellow arrowheads indicate the VLPs. **(C)** The number of VLPs in each image was counted (6–10 images). The P values were determined based on the student's *t*-test. Where, ^***^*P* < 0.0001.

Supplementary Figure 9 The cell morphology was abnormal in the cleaved caspase-3-positive cells. The cleaved caspase-3 positive cells and HERV-K Gag positive cells were analyzed by flow cytometry **(left panels)**. The FSC and SSC in each gate area (Q1–Q4) were shown in the **right panels**.

Supplementary Figure 10 Z-DEVD-FMK and Ac-DEVD-CHO, with a concentration of 10 μM and 2 μM, respectively, have no cytotoxicity in NCCIT and 293T cells. The cell viability was analyzed using an MTT assay. The relative percentage of live cells was shown. For statistically significant analysis, the data from three independent experiments are shown as mean ± standard deviations. In the case of all experiments in this section, the *P* values were determined based on the student's *t*-test. n.s., not significant.

Supplementary Figure 11 The abnormal cell number was reduced by anti-apoptotic drugs in HERV-K Gag-expressing cells. The cleaved caspase-3 positive cells and HERV-K Gag positive cells were analyzed by flow cytometry (left panels) in NCCIT **(A)** and 293T **(B)** cells. The FSC and SSC in each gate area (Q1-Q4) were shown in the right panels.

Supplementary Table 1 The primer information for the PCR.

Supplementary Table 2 Number of VLPs/cell.
