# Supplementary figures and images for: Activation of human endogenous retroviruses by Sox proteins induces cell apoptosis via the caspase-3 pathway
Source: Front Microbiol. 2025 Sep 4;16:1604022. doi: 10.3389/fmicb.2025.1604022 (PMC12443834; doi:10.3389/fmicb.2025.1604022)

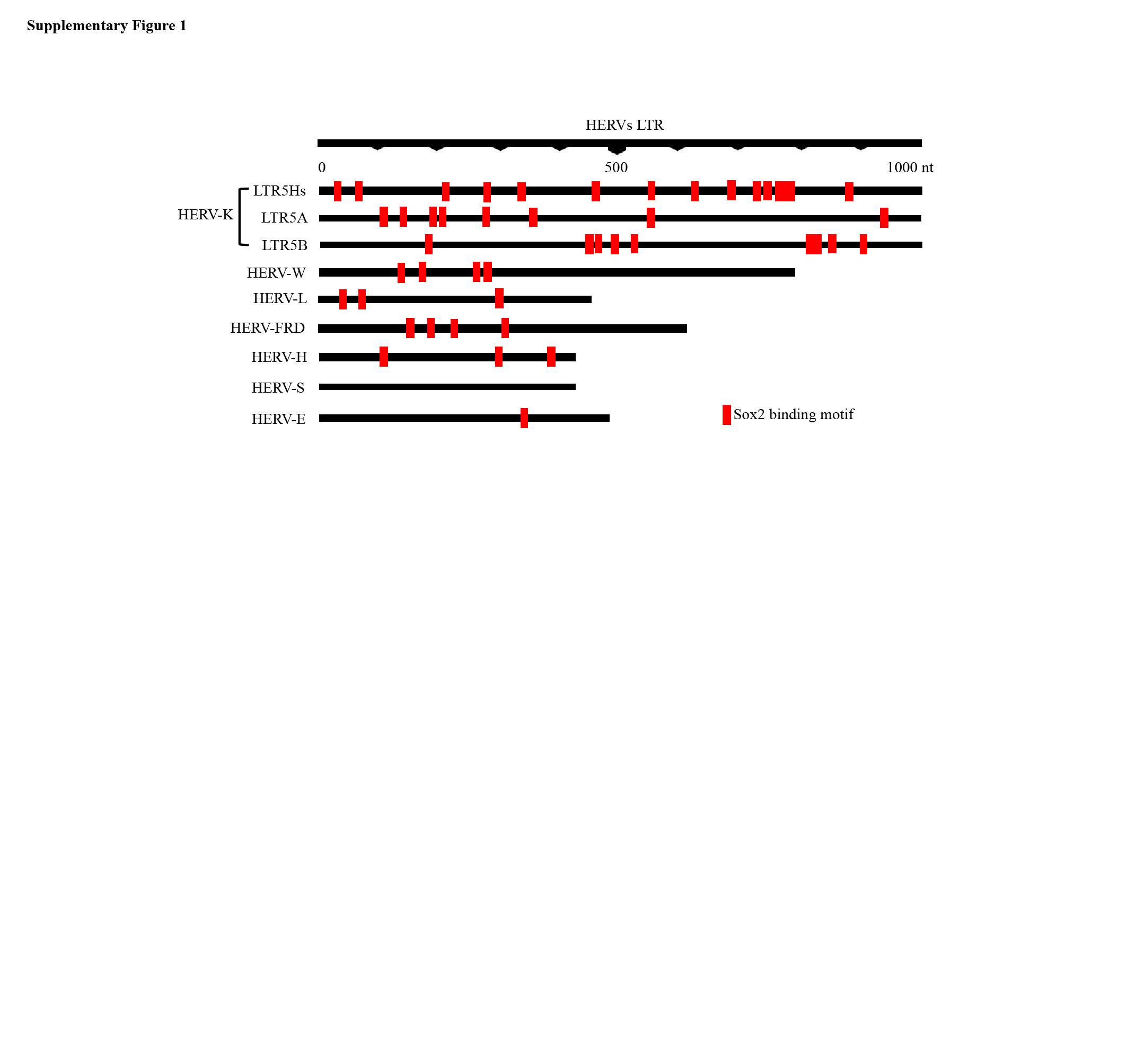

Supplement: Supplementary Figure 1 — Sox binding motifs in LTRs. Except for HERV-S LTR, multiple Sox2 binding motifs were presented in HERV-K LTR5Hs, LTR5A, and LTR5B, HERV-W, L, FRD, H, and E LTR. Red lines indicate the Sox2-binding motifs. [file Image_1.jpeg]

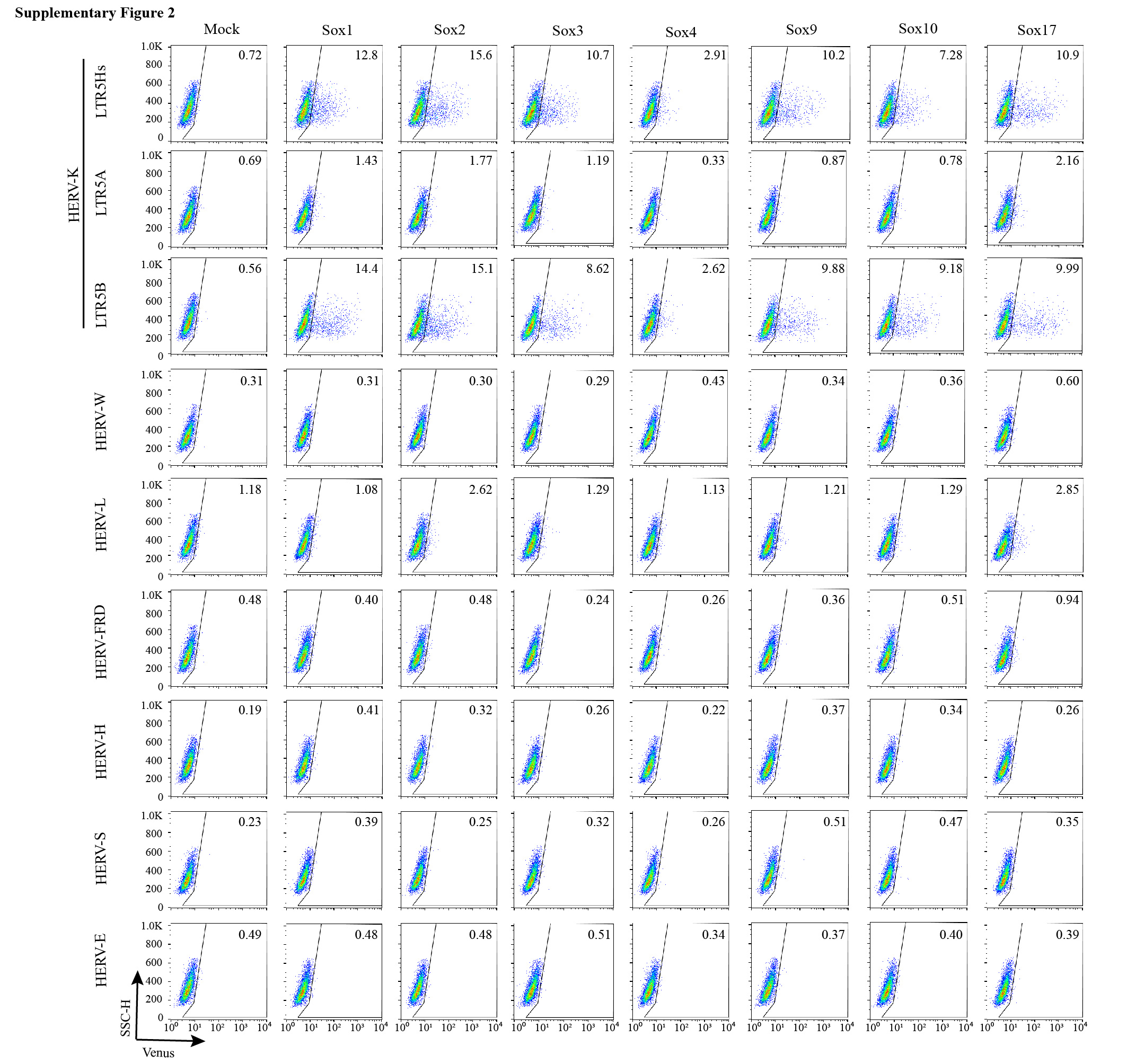

Supplement: Supplementary Figure 2 — Sox proteins activated the HERV-K LTR5Hs and LTR5B in HeLa cells. Several pHERV LTR-Venus were cotransfected with the pMXs-Sox into HeLa cells. At 48 h post-transfection, HERV-K LTR5Hs, LTR5A, and LTR5B, HERV-W, L, FRD, H, S, and E LTR-driven venus positive signal was determined by flow cytometry. [file Image_2.jpeg]

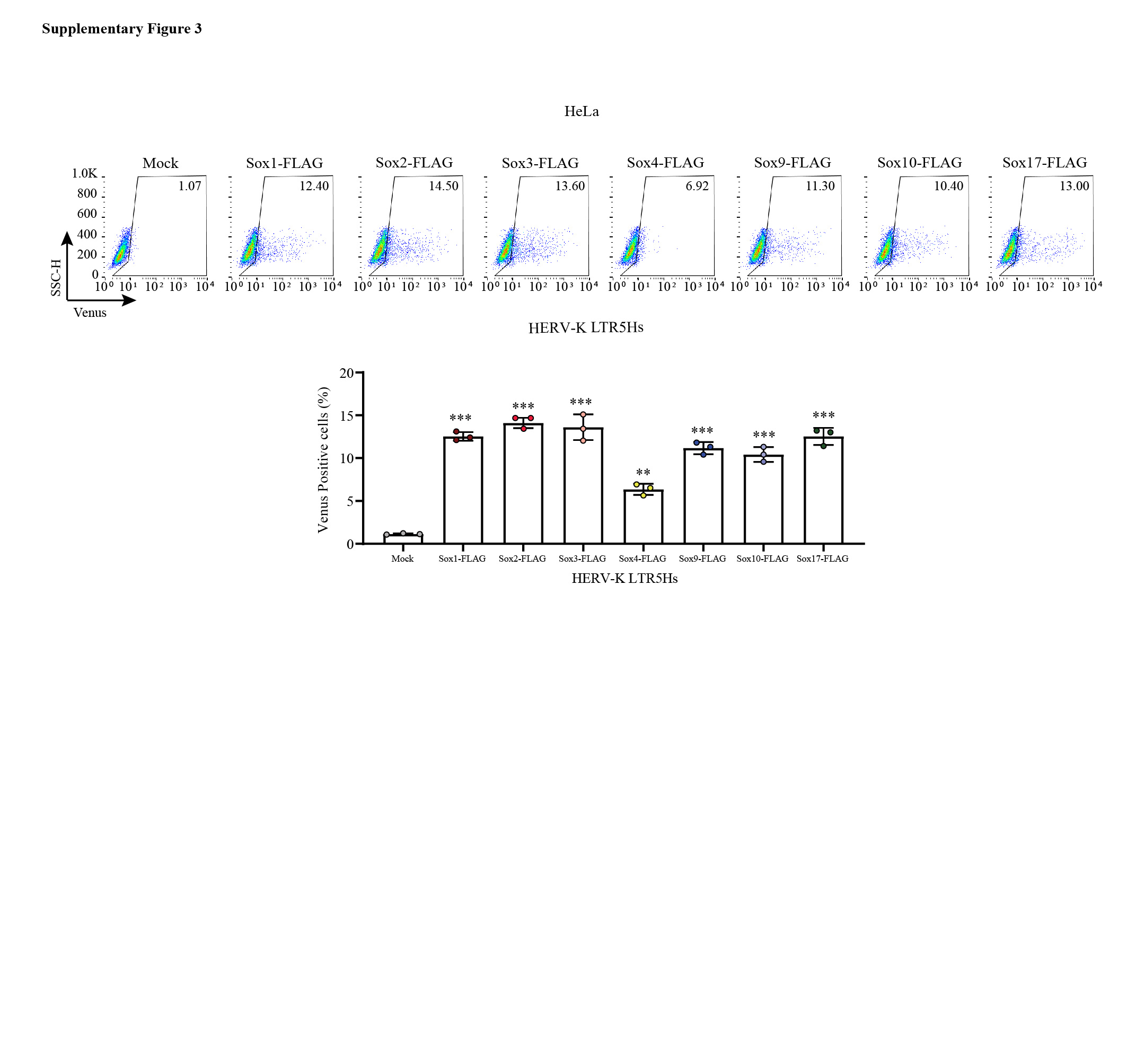

Supplement: Supplementary Figure 3 — The Sox-FLAG proteins activated the HERV-K LTR5Hs activation. Sox-FLAG plasmids were cotransfected with HERV-K LTR5Hs-Venus plasmid into HeLa cells. At 48 h post-transfection, Venus-positive cells were detected by flow cytometry. For statistically significant analysis, the data from three independent experiments are shown as mean ± standard deviations. The P values were determined based on the student's t-test. Where, **P < 0.001; ***P < 0.0001. [file Image_3.jpeg]

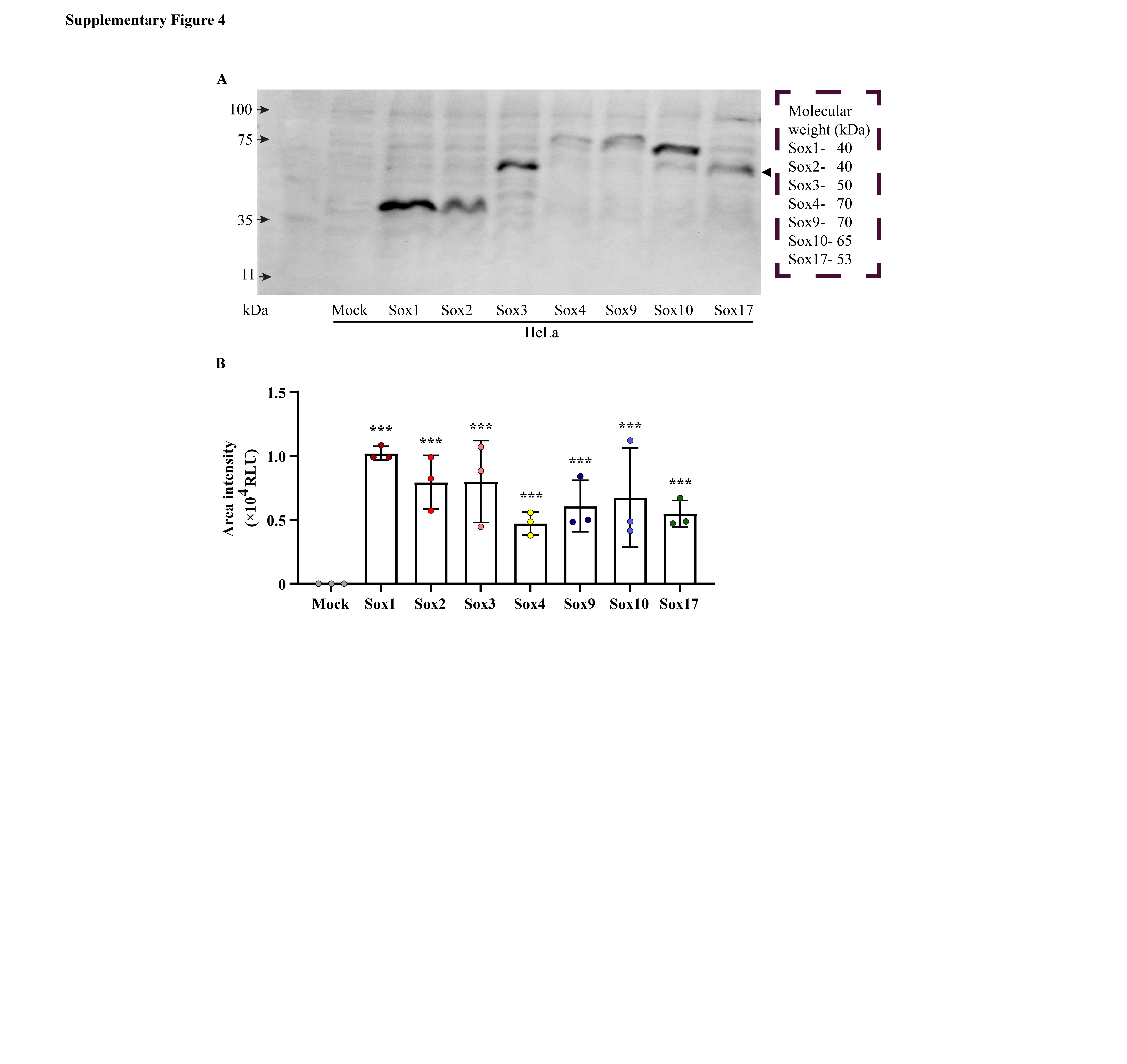

Supplement: Supplementary Figure 4 — The expression of Sox proteins in Sox-FLAG overexpressing HeLa cells. Sox-FLAG plasmids were transfected into HeLa cells. At 48 h post-transfection, the cell supernatants were collected. The molecular weight of each Sox was determined by staining with anti-FLAG antibody. Each molecular weight is shown in the right dot line box (Matsuba et al., 2002). The results are confirmed based on three independent experiments. [file Image_4.jpeg]

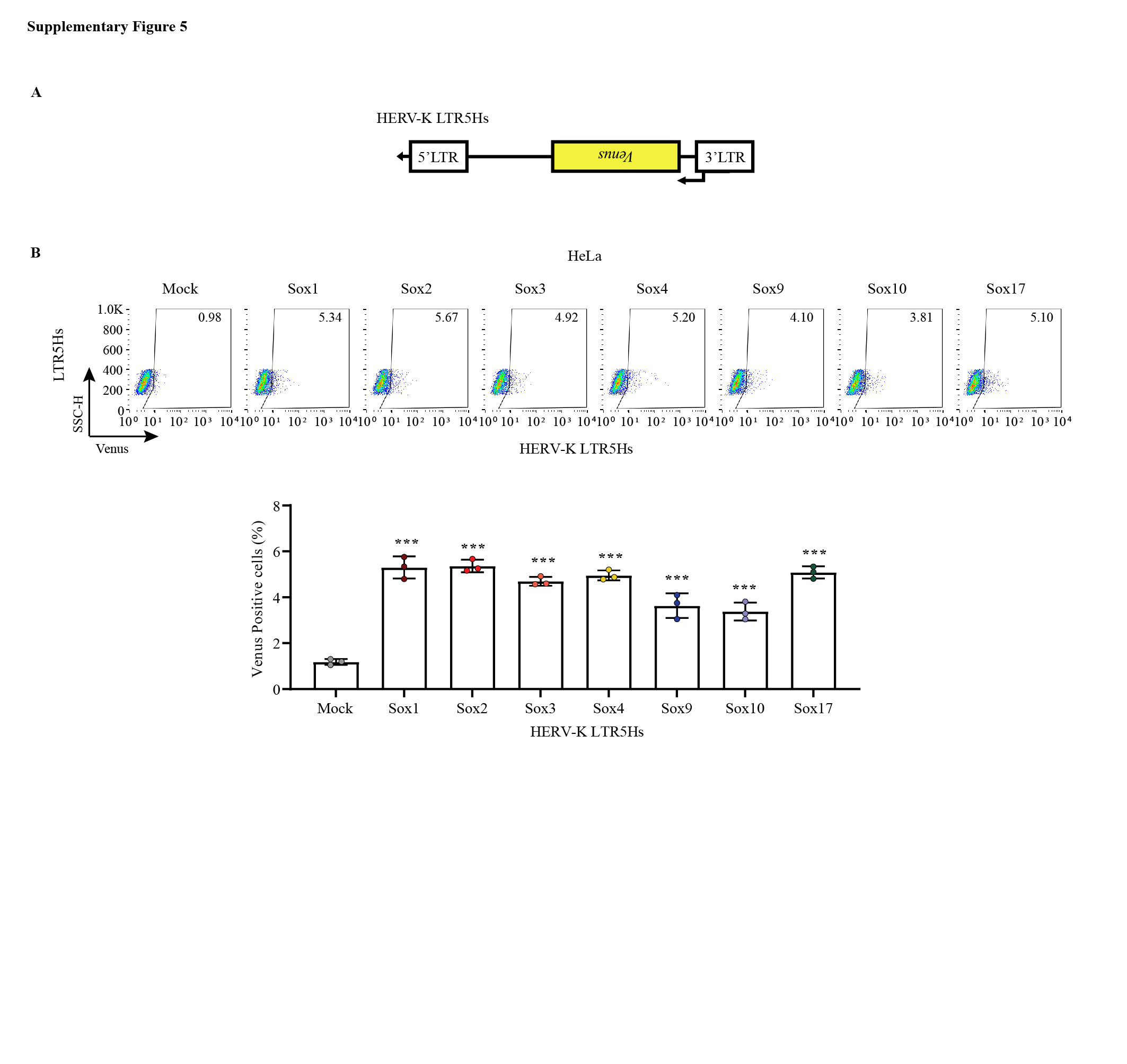

Supplement: Supplementary Figure 5 — Sox proteins activated HERV-K LTR5Hs from the antisense strand. (A) pHERV-K LTR5Hs 3'LTR-driven Venus plasmids were constructed. (B) pHERV-K LTR5Hs-Venus reverse orientation plasmid was cotransfected with pMXs-Sox into HeLa cells. At 48 h post-transfection, Venus-positive cells were detected by flow cytometry. For statistically significant analysis, the data from three independent experiments are shown as mean ± standard deviations. The P values were determined based on the student's t-test. Where, **P < 0.001; ***P < 0.0001. [file Image_5.jpeg]

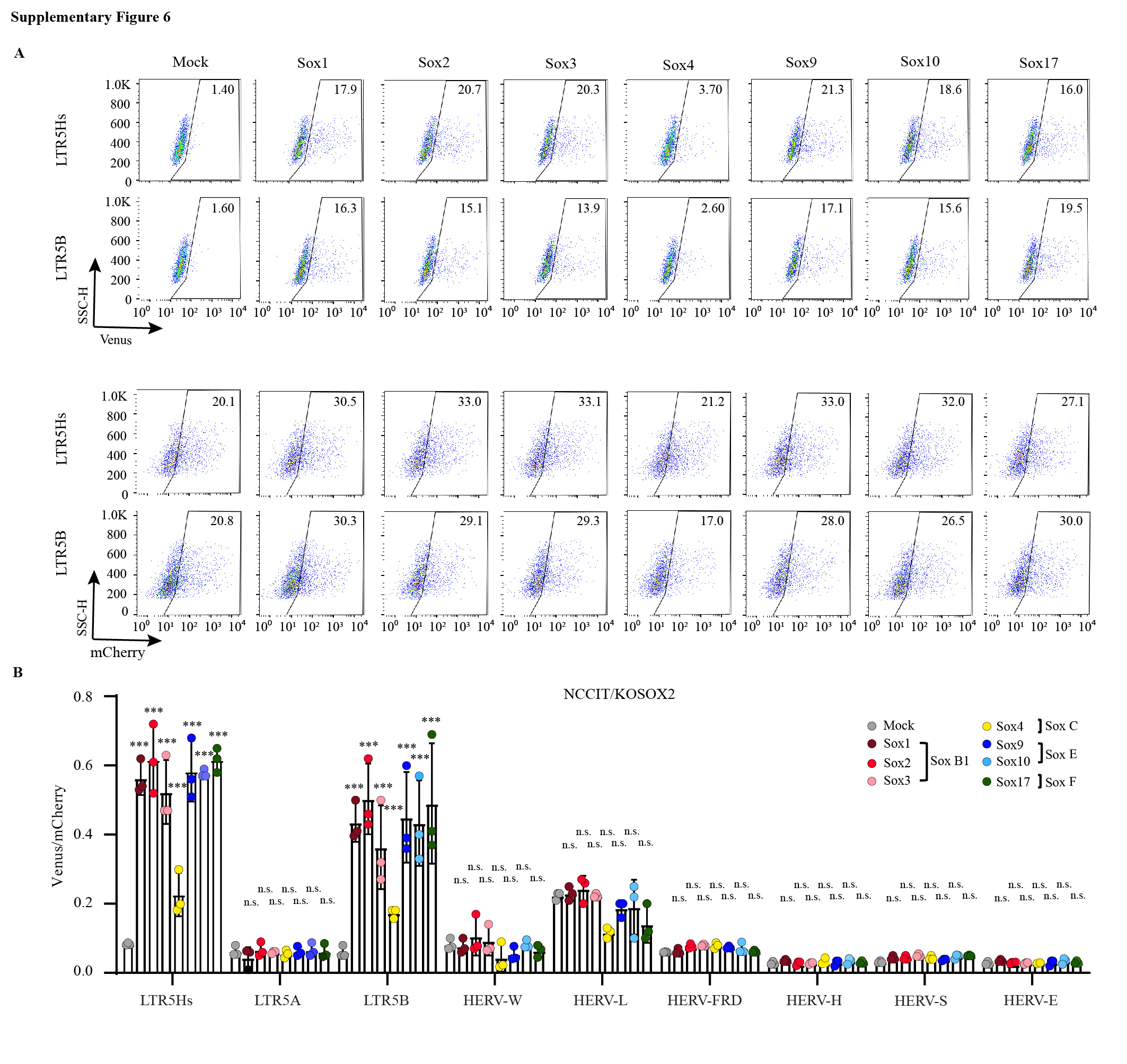

Supplement: Supplementary Figure 6 — HERV-K LTR5Hs and LTR5B were activated by Sox proteins in NCCIT/KOSOX2 cells. (A) Several pHERV LTR-Venus were cotransfected with the pMXs-Sox and pmCherry-N1 into NCCIT/KOSOX2. As internal control to normalize the transfection efficiency, the mCharry-expressing vector (pmCherry-N1) was cotransfected. The Venus and mCherry positive signals were determined by flow cytometry. (B) The Venus positive cell number was divided by the mCherry positive cell number. For statistically significant analysis, the data from three independent experiments are shown as mean ± standard deviations. According to the student's t-test, the P values were determined where ***P < 0.0001; n.s., not significant. [file Image_6.jpeg]

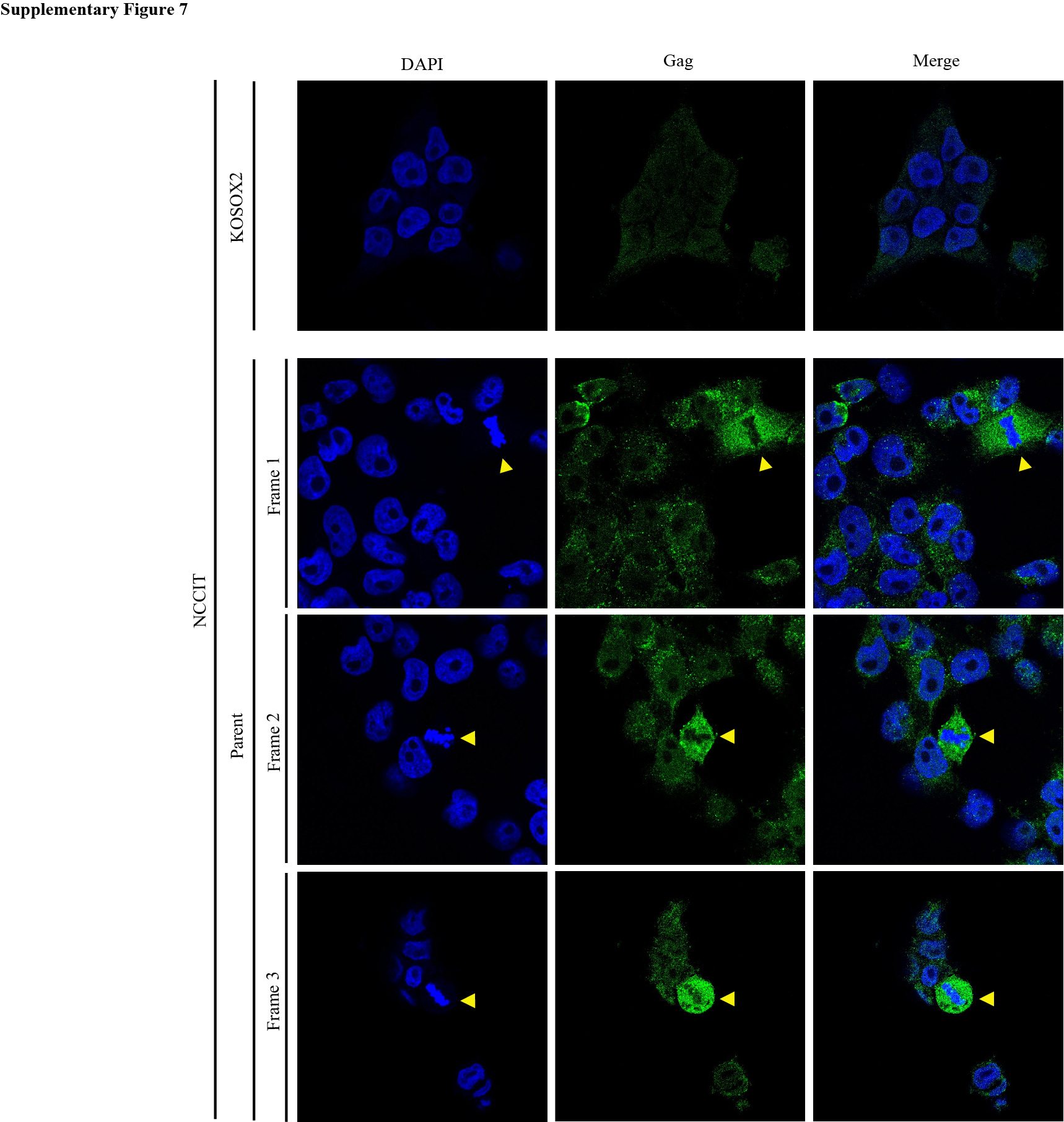

Supplement: Supplementary Figure 7 — The HERV-K Gag was highly expressed in NCCIT cells during cell division, as reported previously (Yamashita et al., 2020). The nucleus was stained with DAPI. The samples were subjected to stain with anti-HERV-K Gag antibody followed by Alexa Flour 488 and observed by confocal microscopy. The arrowhead indicates the expression of HERV-K Gag in dividing NCCIT cells (Frame 1, 2, and 3). The scale bar showed the size of the images. [file Image_7.jpeg]

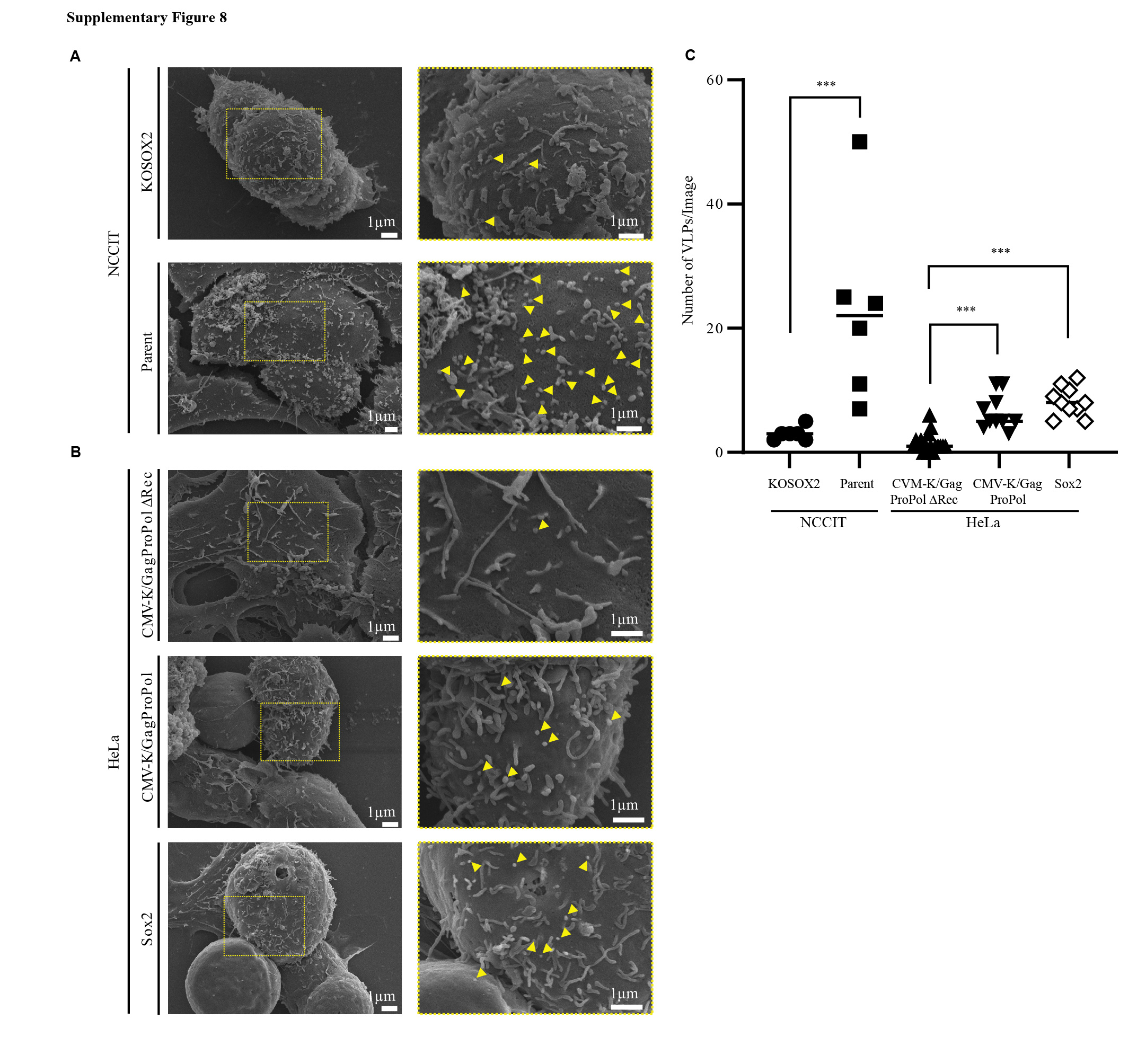

Supplement: Supplementary Figure 8 — The number of VLPs were increased by Sox2 expression. (A) The VLP on the cell surface was observed by scanning electron microscopy in NCCIT/KOSOX2 and NCCIT cells. (B) The VLP on the cell surface was observed by scanning electron microscopy in pCMV-K/GagProPol?Rec, pCMV-K/GagProPolΔRec, and pMXs-Sox2-transfected 293T cells. The expanded images gated by yellow boxes show the right side. The scale bar showed the size of the images. The yellow arrowheads indicate the VLPs. (C) The number of VLPs in each image was counted (6–10 images). The P values were determined based on the student's t-test. Where, ***P < 0.0001. [file Image_8.jpeg]

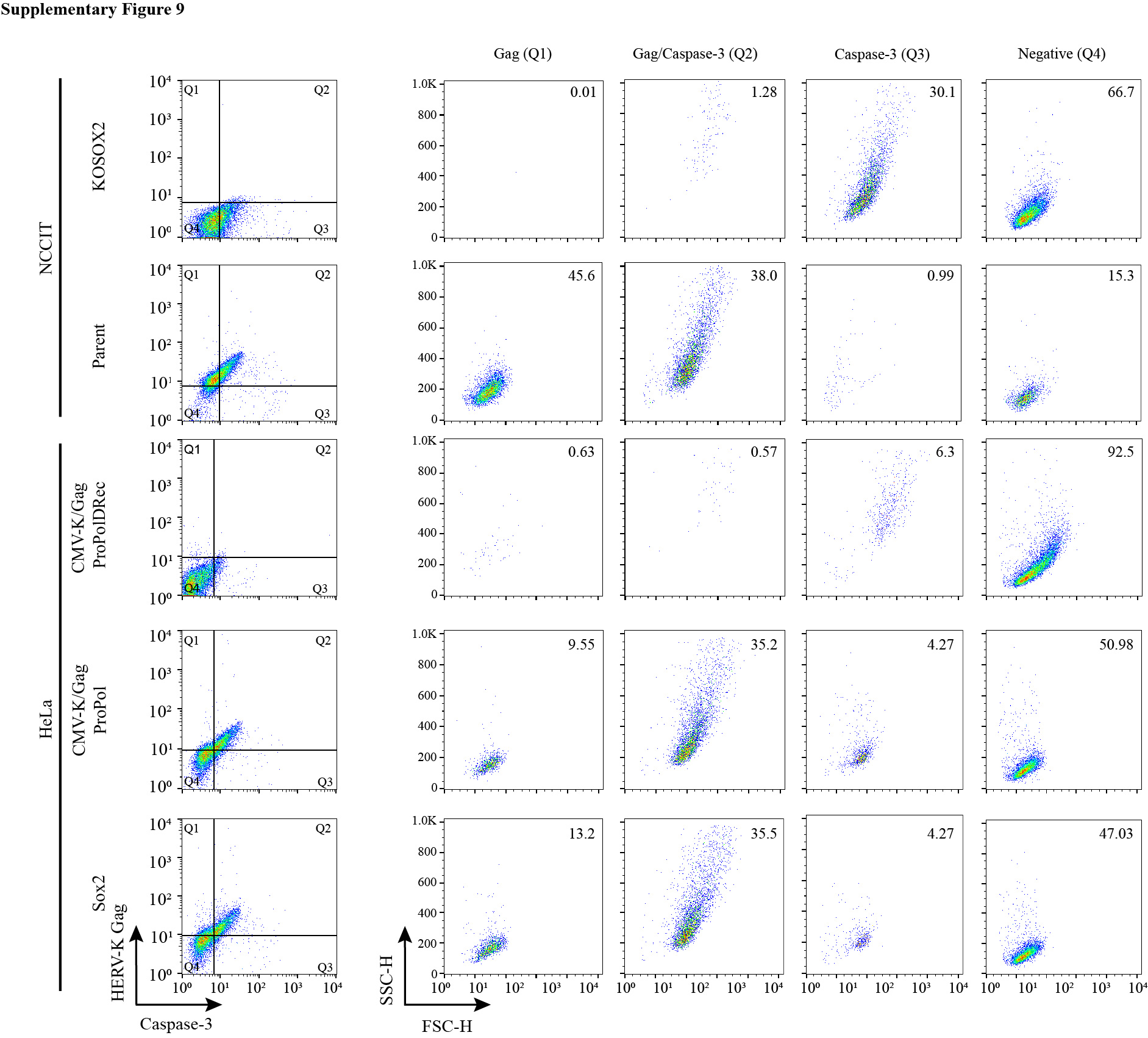

Supplement: Supplementary Figure 9 — The cell morphology was abnormal in the cleaved caspase-3-positive cells. The cleaved caspase-3 positive cells and HERV-K Gag positive cells were analyzed by flow cytometry (left panels). The FSC and SSC in each gate area (Q1–Q4) were shown in the right panels. [file Image_9.jpeg]

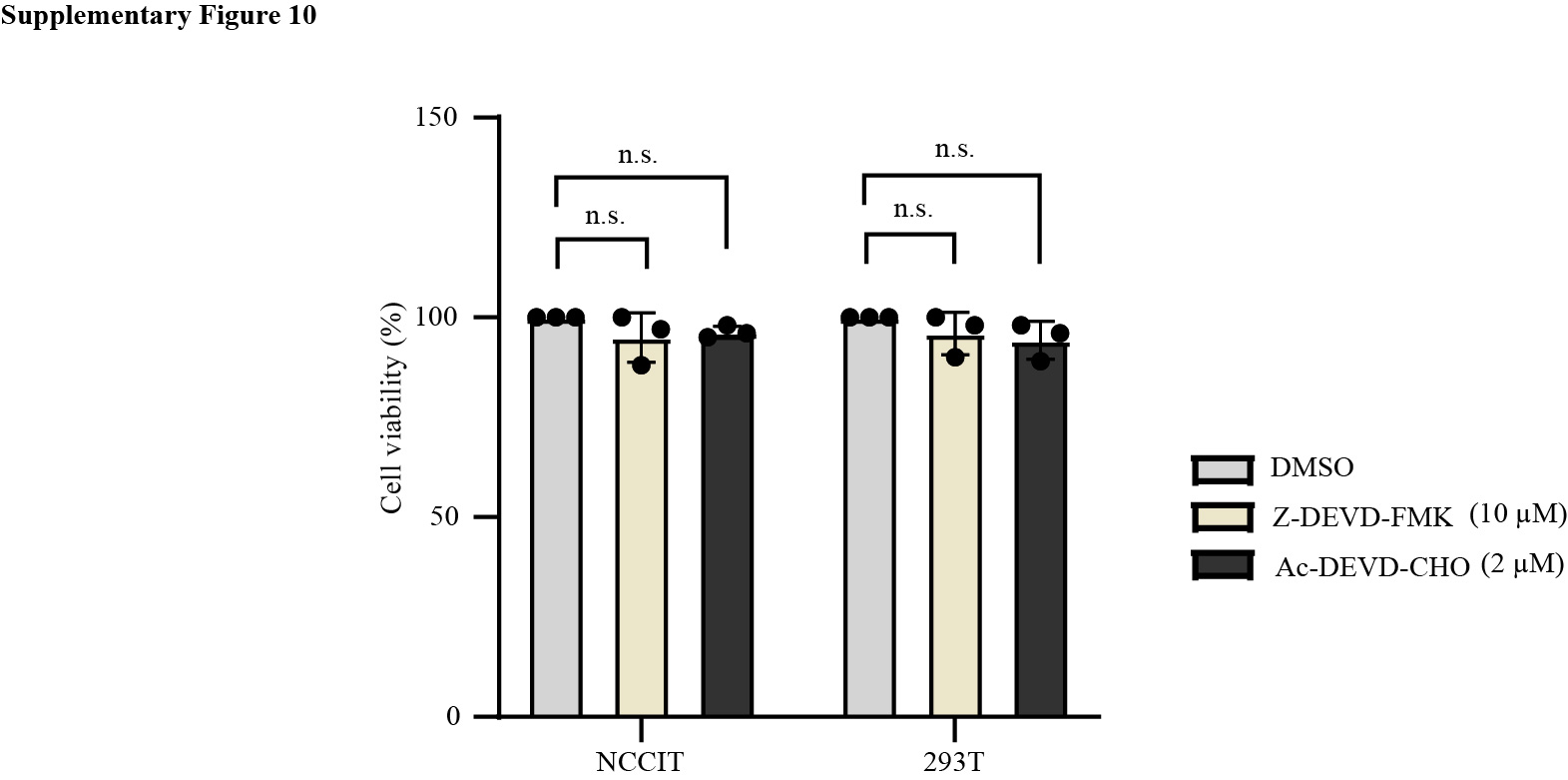

Supplement: Supplementary Figure 10 — Z-DEVD-FMK and Ac-DEVD-CHO, with a concentration of 10 μM and 2 μM, respectively, have no cytotoxicity in NCCIT and 293T cells. The cell viability was analyzed using an MTT assay. The relative percentage of live cells was shown. For statistically significant analysis, the data from three independent experiments are shown as mean ± standard deviations. In the case of all experiments in this section, the P values were determined based on the student's t-test. n.s., not significant. [file Image_10.jpeg]

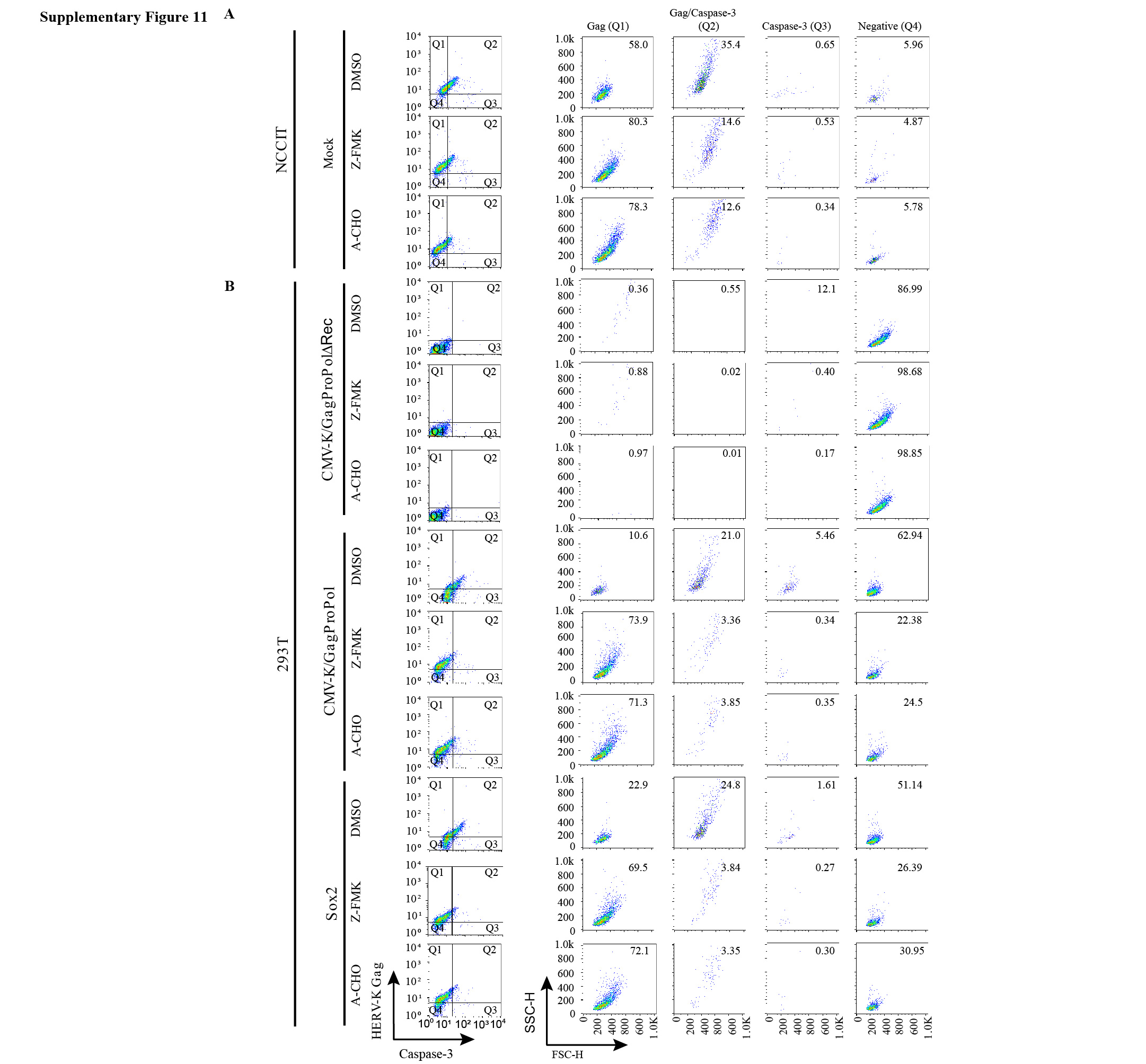

Supplement: Supplementary Figure 11 — The abnormal cell number was reduced by anti-apoptotic drugs in HERV-K Gag-expressing cells. The cleaved caspase-3 positive cells and HERV-K Gag positive cells were analyzed by flow cytometry (left panels) in NCCIT (A) and 293T (B) cells. The FSC and SSC in each gate area (Q1-Q4) were shown in the right panels. [file Image_11.jpeg]
