# Supplementary material for: Activation of human endogenous retroviruses by Sox proteins induces cell apoptosis via the caspase-3 pathway
Source: Front Microbiol. 2025 Sep 4;16:1604022. doi: 10.3389/fmicb.2025.1604022 (PMC12443834; doi:10.3389/fmicb.2025.1604022)
Supplement: Supplementary Table 1 — The primer information for the PCR. [file Table_1.docx]

| **Name of primers** | **Orientation** | **Sequence (5’-3’)** |
| --- | --- | --- |
| HERV-K LTR5Hs | Forward | CCTTGCATGGGGAGATTCAG |
|  | Reverse | GCCTCCACGTTGGGCACCAG |
| HERV-K LTR5A | Forward | TAATCAGTAAAAACTGAGGG |
|  | Reverse | CTCCACGTTGGGCACCAG |
| HERV-K LTR5B | Forward | CCTATGACCCTGCCACATCC |
|  | Reverse | ATAGGCGTATCACGAGGCCC |
| HERV-W LTR | Forward | TGAGAGACAGGACTAGCTG |
|  | Reverse | GCCAAAATGTTACCGGGGGG |
| HERV-L LTR | Forward | TGTGATGATTAATACCAAGTCG |
|  | Reverse | TGTATTACTCAAGGTTCTCT |
| HERV-FRD LTR | Forward | CACTAGGGAAGGTATCCGAGTC |
|  | Reverse | AGCGAGGCCTGTCTCCAAAA |
| HERV-H LTR | Forward | TGTCAGGCCTCTGAGCCCAA |
|  | Reverse | TTTCATGTGCGTCCATGTGA |
| HERV-S LTR | Forward | GTGCAGCCAGGCAGGCATAG |
|  | Reverse | ATGTCAGGGTCAGAGTCCCC |
| HERV-E LTR | Forward | GAACTGTCGTTTAAACACCC |
|  | Reverse | GCCAGAGTCCCCCACAGGGA |
| Sox1 | Forward | ACGGGAATTCGCCCTTATGTACAGCATGATGATGGA |
|  | Reverse | GACTGAC TAAGCTTGATGTGCGTCAGGGGCACCG |
| Sox2 | Forward | CAGTGTGGTGGTAC GGGAATTCGCCCTATGTACAA |
|  | Reverse | TTTATAATCAAGCTTCATGTGTGAGAGGGGCAGTG |
| Sox3 | Forward | ACGGGAATTCGCCCTTATGCGACCTGTTCGAGAGAA |
|  | Reverse | GACTGACTAAGCTTGATGTGGGTCAGCGGCACCG |
| Sox4 | Forward | ACGGGAATTCGCCCTTATGGTGCAGCAAACCAACAA |
|  | Reverse | GACTGACTAAGCTTGTAGGTGAAAACCAGGTTGG |
| Sox9 | Forward | ACGGGAATTCGCCCTTATGAATCTCCTGGACCCCTT |
|  | Reverse | GACTGACTAAGCTTGGGTCTGGTGAGCTGTGTGT |
| Sox10 | Forward | ACGGGAATTCGCCCTTATGGCGGAGGAGCAGGACCT |
|  | Reverse | GACTGACTAAGCTTGGGCCGG GACAGTGTCGTAT |
| Sox17 | Forward | ACGGGAATTCGCCCTTATGAGCAGCCCGGATGCGGG |
|  | Reverse | GACTGACTAAGCTTCACGTCAGGATAGTTGCAGT |
| HERV-K gag | Forward | GTTTCAGTTTCTGATGCCCC |
|  | Reverse | GTTTAGACTCTGATGGCCCC |
| LTR-F1 | Forward | TTGTGGGGAAAAGCAAGA |
|  | Reverse | CTGTTTAACAAAGCACATCC |
| LTR-F2 | Forward | ATGCTTGAAGGCAGCATGCT |
|  | Reverse | CCTCAGCACAGACCCTTTAC |
| LTR-F3 | Forward | AGGATTAGTATAAGAGGAAG |
|  | Reverse | GGATATGCATACACATAAAC |
| LTR-F4 | Forward | AAAAGCACAGCACTTAATCC |
|  | Reverse | CCGGGGAACCAGCGTTCAGC |
| LTR-F5 | Forward | ATACTAAGGGAACTCAGAGG |
|  | Reverse | AGGTGGGATGAGAGATTTGG |
| GAPDH | Forward | CAAGAATGGTGAAGCAGG |
|  | Reverse | ACAAAGTGGTCGTTGAGGGC |

**Supplementary Table 1.**
