# Supplementary material for: Activation of human endogenous retroviruses by Sox proteins induces cell apoptosis via the caspase-3 pathway
Source: Front Microbiol. 2025 Sep 4;16:1604022. doi: 10.3389/fmicb.2025.1604022 (PMC12443834; doi:10.3389/fmicb.2025.1604022)
Supplement: Supplementary Table 2 — Number of VLPs/cell. [file Table_2.docx]

**Supplementary Table 2.**

| **Number of cell count** | **Number of VLPs/cell** | | | | |
| --- | --- | --- | --- | --- | --- |
|  | **NCCIT/KOSOX2** | **NCCIT** | **HeLa** | | |
|  |  |  | pCMV-K/GagProPolΔRec | pCMV-K/GagProPol | pMXs-Sox2 |
| 1 | 2 | 25 | 1 | 7 | 7 |
| 2 | 3 | 7 | 0 | 5 | 10 |
| 3 | 5 | 50 | 1 | 5 | 11 |
| 4 | 3 | 24 | 1 | 11 | 12 |
| 5 | 3 | 20 | 0 | 5 | 9 |
| 6 | 2 | 11 | 1 | 4 | 5 |
| 7 |  |  | 2 | 3 | 5 |
| 8 |  |  | 2 | 4 | 8 |
| 9 |  |  | 1 | 5 | 9 |
| 10 |  |  | 0 | 11 | 7 |
| 11 |  |  | 3 |  |  |
| 12 |  |  | 1 |  |  |
| 13 |  |  | 4 |  |  |
| 14 |  |  | 3 |  |  |
| 15 |  |  | 1 |  |  |
| 16 |  |  | 1 |  |  |
| 17 |  |  | 1 |  |  |
| Average | 3.0 | 22.8 | 1.4 | 6.0 | 8.3 |
| Stdev | 1.0 | 14.4 | 0.5 | 2.4 | 2.5 |
